# Formation and Rearrangement of a Congested Spiropentane from the Trapping of Dibenzonorcarynyliden(e/oid) by Phencyclone

**DOI:** 10.1021/acs.orglett.4c01001

**Published:** 2024-04-26

**Authors:** Alexander D. Roth, Dasan M. Thamattoor

**Affiliations:** Department of Chemistry, Colby College, Waterville, Maine 04901, United States

## Abstract

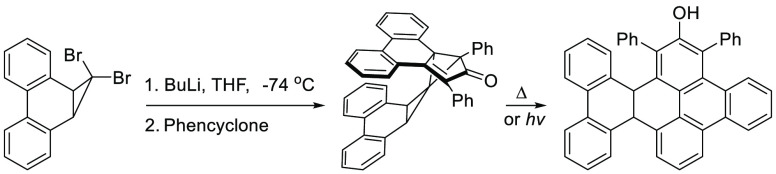

The low-temperature treatment of 1,1-dibromo-1a,9b-cyclopropa[*l*]phenanthrene with butyllithium appeared to produce dibenzonorcarynyliden(e/oid) which could be intercepted with phencyclone to produce a hindered spiropentane. The spiropentane readily rearranges, thermally and photochemically, into a triphenylene phenol derivative. The spiropentane and its rearrangement product were characterized by X-ray crystallography.

Several years ago, we reported that the Doering–Moore–Skattebøl (D–M–S) reaction^1−3^ of 1,1-dibromo-1a,9b-cyclopropa[*l*]phenanthrene (**1**) with butyllithium and copper(II) chloride in THF gave the hydrocarbon **2**, a cyclotetramer of the strained allene dibenzocycloheptatetraene (**5**), as shown in Scheme 1.^4^ Also formed as a byproduct in this reaction is the ether **6** which could be rationalized as an insertion reaction of dibenzonarcarnylidene (**4**), or a carbenoid equivalent **3** that qualitatively behaves like the carbene,^5,6^ with the THF used as solvent. We have also previously reported the ylide-forming reaction of **4** with THF.^7^

**Scheme 1:**
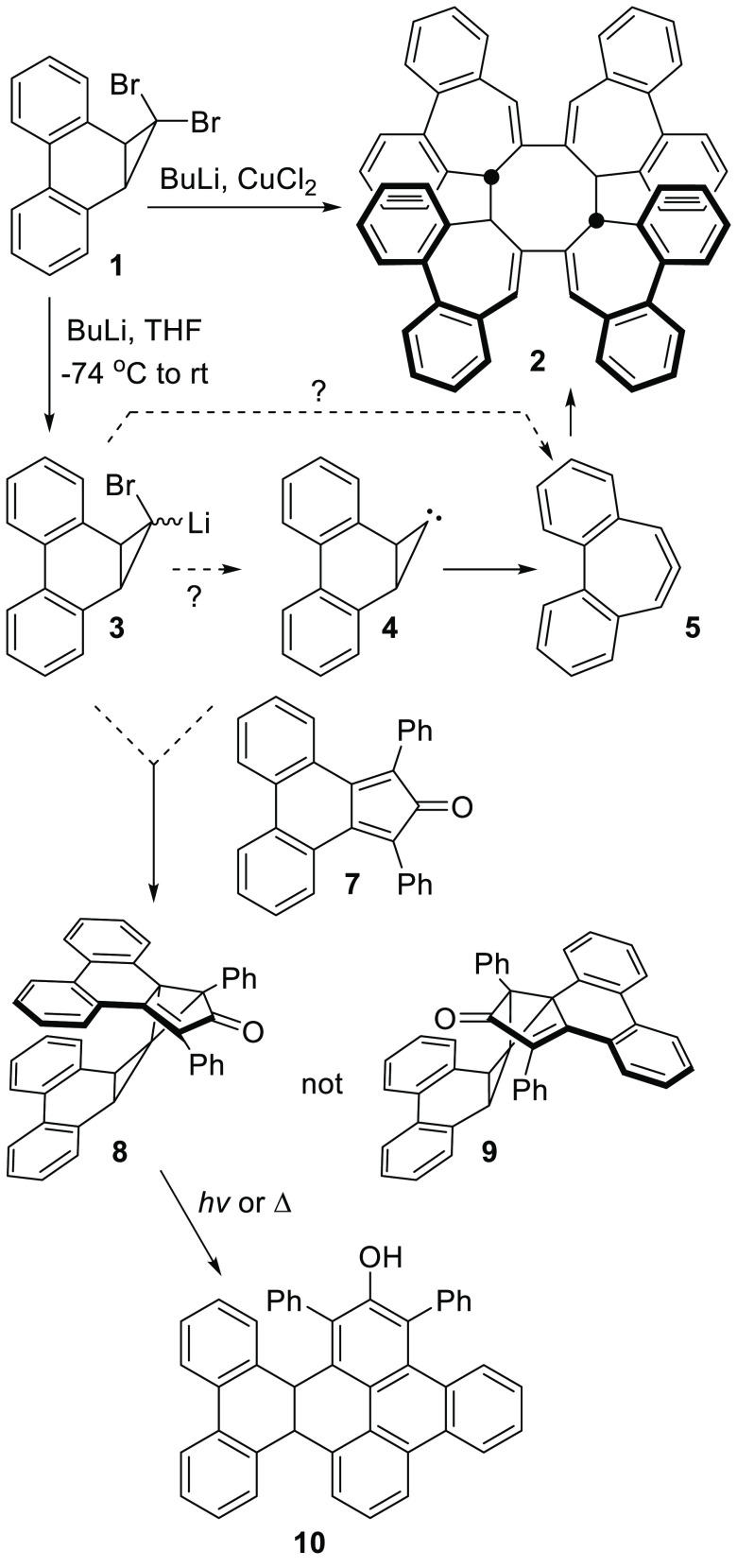
Generation of a Formal Tetramer of Dibenzocycloheptatetraene That Was Previously Reported^4^ and the Interception of Dibenzonorcarynyliden(e/oid) with Phencyclone to Form a Congested Spiropentane Which Subsequently Rearranges, Thermally or Photochemically, to a Triphenylene Phenol (This Work)

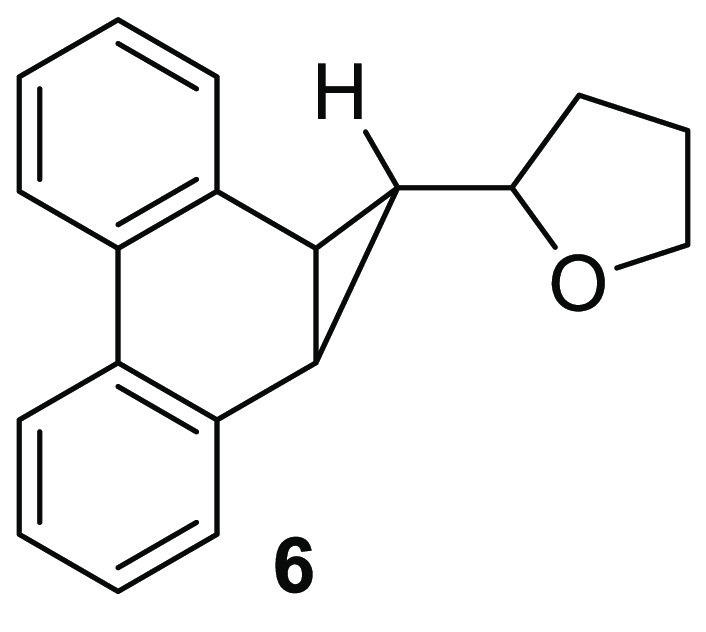



Herein, we describe a congested spiropentane **8** formed in the reaction of **1** with butyllithium at low temperature in the presence of phencyclone (**7**) as also depicted in Scheme 1. It is notable that **8**, with the phenanthrene ring *endo* to the biphenyl moiety, is produced exclusively, with no evidence for the formation of the *exo* diastereomer **9**. Spiropentane **8** appears to be rather unstable and rearranges almost quantitatively into the phenol **10** upon exposure to heat or light. Interestingly, the color of **10** changed based on the solvent in which the rearrangement occurred. Specifically, thermal rearrangement in hexanes afforded **10** as a red solid that precipitated out of solution, while thermolysis in chloroform or photolysis in benzene-*d*_6_ afforded a colorless solid. Dissolving red **10** in dichloromethane irreversibly removed the red color (see the Supporting Information). Single-crystal X-ray structures of **8** and the red form of **10** are shown in Figure 1. Crystals suitable for X-ray diffraction could not be obtained from the colorless solid. The FTIR spectra of the red and colorless forms of **10**, however, appeared to be identical. One plausible explanation for these observations is that the red and colorless forms of **10** have a polymorphic relationship. Polymorphs can have sharply different colors,^8^ and our laboratory has also reported such an occurrence previously.^9^

**Figure 1:**
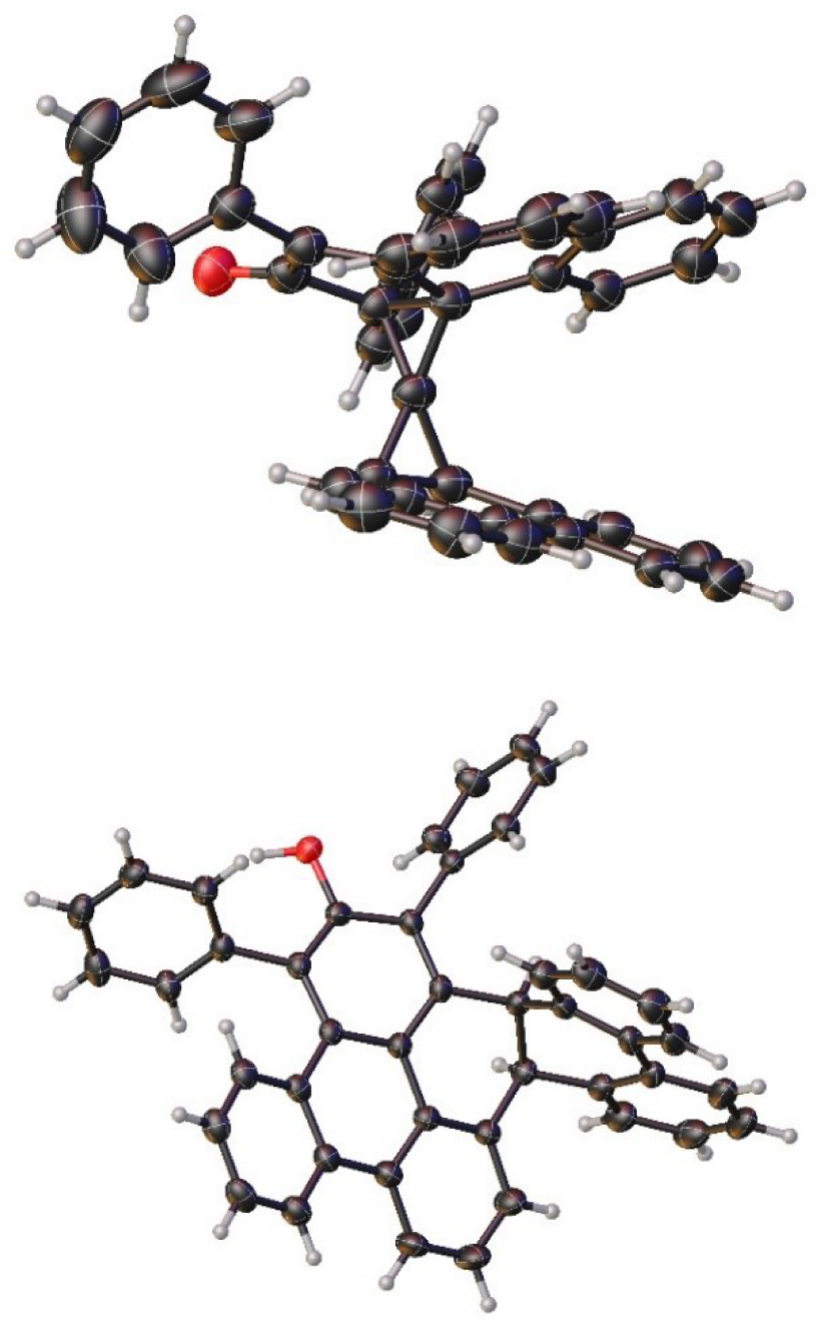
Single-crystal X-ray structures of **8** (top) and **10** (bottom). Thermal ellipsoids are shown at the 50% probability level.

Formation of **8** may be explained by the direct addition of **4** to **7** as shown in Scheme 1. An alternative possibility is the Michael addition of **3** to **7** to give the enolate **11** followed by an intramolecular nucleophilic displacement of bromide within **11** to form **8** (Scheme 2). Given the difficulty in effecting nucleophilic substitution reactions on cyclopropyl substrates,^10^ the mechanism shown in Scheme 2 was deemed unlikely, however, as it requires a hindered nucleophile to displace bromide from a tertiary center in an S_N_2-like process with an inversion of configuration at a cyclopropyl carbon.^11^

**Scheme 2:**
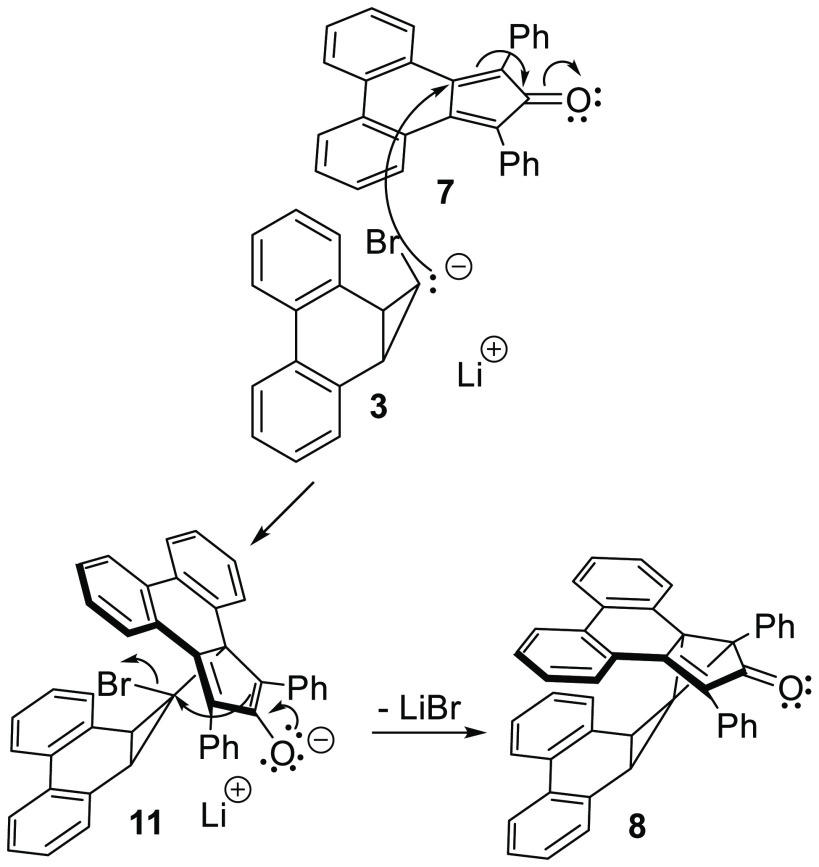
An Alternative, but Unlikely, Mechanism for the Formation of **8** Initiated by a Michael Reaction

Although the precise manner by which **8** rearranges into **10** remains unknown, a plausible mechanistic hypothesis is offered in Scheme 3. The first step, which simultaneously aromatizes the central phenanthrene ring of the erstwhile phencyclone *and* relieves strain in the spiropentane moiety, leads to the zwitterion **12**. It is notable that the anionic part of the zwitterion is an enolate, whereas the cation enjoys extensive benzylic stabilization. One particular resonance form of **12**, designated as **12′**, may be viewed as a vinylogous cyclopropylcarbinyl cation. Rearrangement of this cation, effectively initiating an electrophilic aromatic substitution, is accompanied by strain release in the cyclopropyl ring and formation of intermediate **13**. Finally, a proton transfer accompanied by aromatization converts **13** into the observed phenol **10**.

**Scheme 3:**
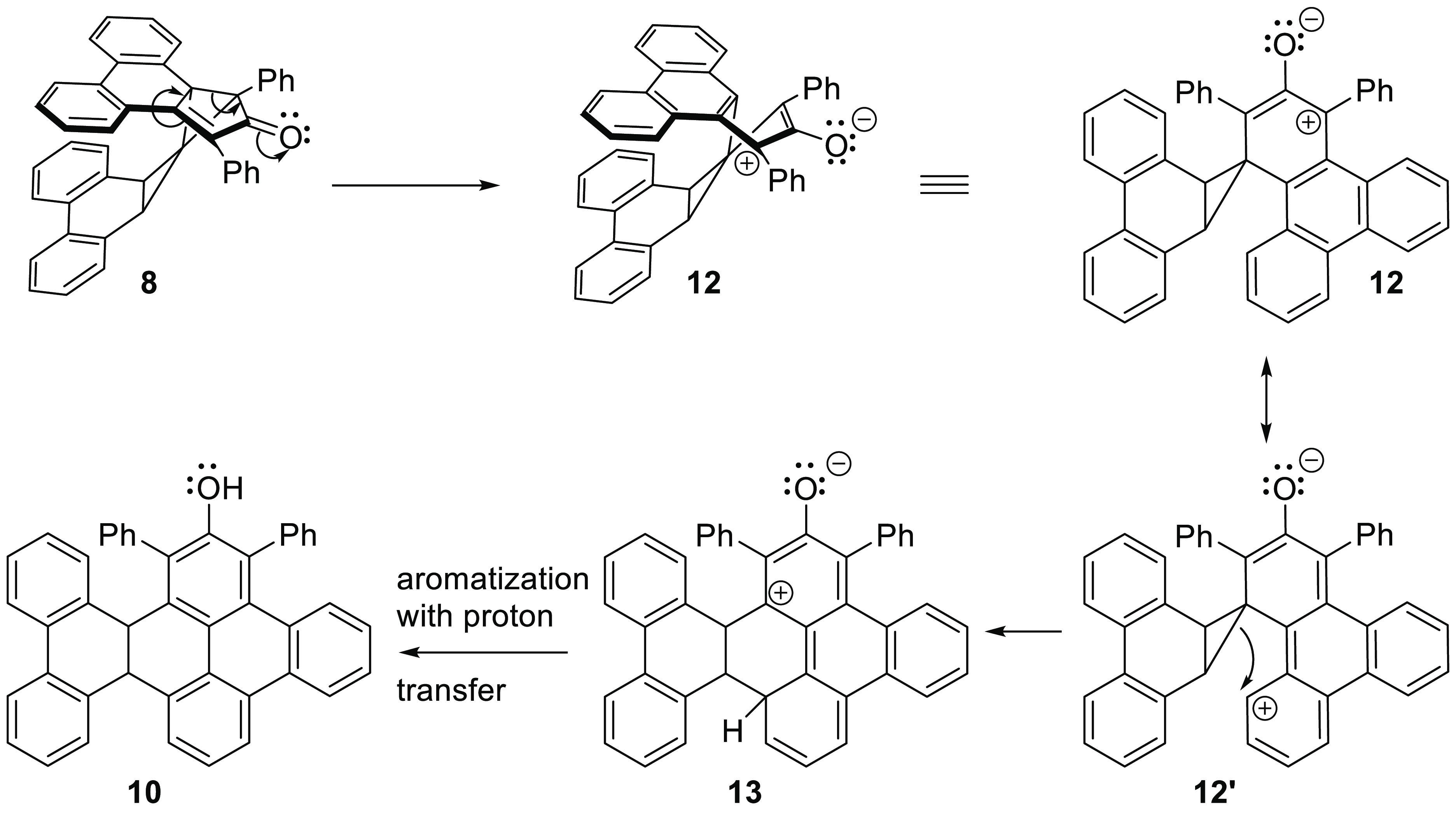
Proposed Mechanism for the Rearrangement of Spiropentane **8** into Phenol **10**

The structure and reactivity of **4** were calculated at the DLPNO-CCSD(T)/def2-TZVP//B2PLYP/def2-TZVP level of theory.^12−19^ These calculations indicated that triplet **4** was 15.40 kcal/mol higher in energy than the singlet (see the Supporting Information).^20^ The calculated potential energy surface (PES) connecting singlet **4** to allene **5** is shown in Figure 2. These calculations revealed that singlet carbene **4** has to overcome a barrier of 6.70 kcal/mol en route to **5**, and that **5** was 49.3 kcal/mol lower in energy than **4**.

**Figure 2:**
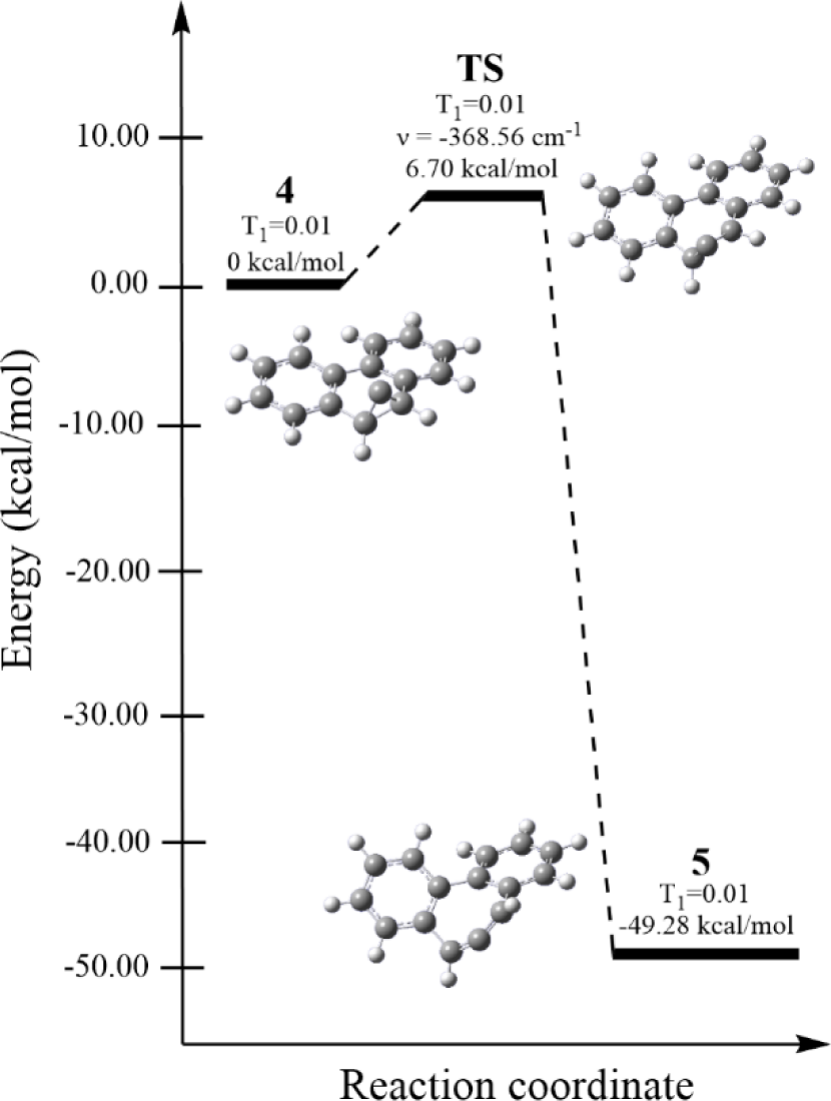
Potential energy surface diagram for the ring opening of singlet **4** to allene **5** through transition state **TS** computed at the DLPNO-CCSD(T)/def2-TZVP//B2PLYP/def2-TZVP level of theory. Optimized geometries, relative energies, imaginary frequency, and *T*_1_ diagnostic values are shown.

Additionally, DLPNO-CCSD(T)/def2-TZVP//B3LYP/def2-SVP calculations^13,14,17−19,21,22^ indicated that the *endo* spiropentane adduct **8** is 6.68 kcal/mol more stable than its *exo* isomer **9**. This preference is consistent with our experimental observations and may be attributable to the favorable π-stacking interactions between the phenanthrene and biphenyl moieties in **8** and the transition state leading up to it. The optimized structures of **8** and **9** are presented in the Supporting Information.

In summary, we have synthesized a crowded *endo* spiropentane **8**, formed via the formal trapping of a dibenzonorcarynyliden(e/oid) (**3/4**) by phencyclone. Computational studies predict that the *endo* diastereomer is more stable than the *exo* form which was not detected as a product. Calculations also show that singlet **4** is more stable than the triplet and has to overcome a modest barrier to ring open to the relatively more stable strained cyclic allene **5**. The light- or heat-induced rearrangement of **8** into phenol **10** was also observed and rationalized by a plausible mechanism.

## Data Availability

The data underlying this study are available in the published article and its Supporting Information.

## Supporting Information Available

Experimental procedures, NMR and IR spectra, crystal structures, and computational details (DOCX)
