# Supplementary material for: Formation and Rearrangement of a Congested Spiropentane from the Trapping of Dibenzonorcarynyliden(e/oid) by Phencyclone
Source: Org Lett. 2024 Apr 26;26(18):3840–3. doi: 10.1021/acs.orglett.4c01001 (PMC11091884; doi:10.1021/acs.orglett.4c01001)
Supplement: Supplementary file 1 — ol4c01001_si_001.docx [file ol4c01001_si_001.docx]

**ELECTRONIC SUPPORTING INFORMATION**

**Formation and Rearrangement of a Congested Spiropentane from the Trapping of Dibenzonorcarynyliden(e/oid) by Phencyclone**

Alexander D. Roth and Dasan M. Thamattoor*

Department of Chemistry, Colby College, Waterville, ME 04901 USA

[*dmthamat@colby.edu](mailto:*dmthamat@colby.edu)

**Table of Contents**

**General experimental procedures**……………………………………………………………..S2

**Computational procedures**…………………………………………………………………….S2

**Synthetic procedures:**

(a) Synthesis of 2',11a'-diphenyl-1a,9b-dihydrospiro[cyclopropa[l]phenanthrene-1,11'-

cyclopropa[1,5]cyclopenta[1,2-l]phenanthren]-1'(11a'H)-one (**8**)………………………...…S3

(b) Synthesis of 9,11-diphenyl-8b,18b-dihydrotetrabenzo[a,c,fg,op]tetracen-10-ol (**10**)….S3-4

**Characterization Data:**

(a) ^1^H NMR of **8**……………………………………………………………………………...S5

(b) ^13^C NMR of **8**……………………………………………………………………………..S6

(c) FTIR spectrum of **8**…………………………………………………………………….....S7

(d) Crystal structure of **8** and salient data ……………………………………………………S8

(e) ^1^H NMR of **10**…………………………………………………………………………….S9

(f) ^13^C NMR of **10**…………………………………………………………………………..S10

(g) FTIR spectrum of **10**………………………………………………………………...…..S11

(h) Crystal structure of **10** and salient data………………………………………………….S12

(i) Gif of red **10** changing color upon solvation in dichloromethane……………………….S13

**Computational Data:**

1. Optimized energies, coordinates, frequencies, and geometry for Singlet **4**; B2PLYP/def2-TZVP………………………………………………………………………………...S14-15
2. Optimized energies, coordinates, frequencies, and geometry for Triplet **4**; B2PLYP/def2-TZVP………………………………………………………………...........................S15-17
3. Optimized energies, coordinates, frequency, and geometry for Allene **5**; B2PLYP/def2-TZVP………………………………………………………………………………...S17-18
4. Optimized energies, coordinates, frequencies, and geometry for transition state **TS**; B2PLYP/def2-TZVP………………………………………………………………...S18-20
5. Single point energies and T1 diagnostics for **4** to **5** PES; CCSD(T)/def2-TZVP//B2PLYP/def2-TZVP……………………………………………………………S20
6. Optimized energies, coordinates, frequencies, and geometry for **9**; B3LYP/def2-SVP…………………………………………………………………………………..S20-23
7. Optimized energies, coordinates, frequencies, and geometry for **8**; B3LYP/def2-SVP…………………………………………………………………………………..S23-26
8. Single point energies and T1 diagnostics for **9** and **8**; CCSD(T)/def2-TZVP//B3LYP/def2-SVP………….……………………………………………………………………..……S26
9. PES diagram for singlet **4** to allene **5**…………………………………………………...S27

**References**………………………………………………………………………………….S27-28

**General Experimental Procedures.**

Tetrahydrofuran was degassed by purging with nitrogen and dried by passage through two activated alumina columns (2ft × 4in). All other solvents and reagents were used as obtained from commercial sources. Unless otherwise noted, all reactions were carried out under an argon atmosphere in oven-dried glassware. The syntheses of 1,1-dibromo-1a,9b-dihydro-1H-cyclopropa[l]phenanthrene (**1**)^1^ and phencyclone (**7**)^2^ were performed according to the literature. Medium pressure flash chromatography was performed on an automated system on pre-packed silica gel columns (70-230 mesh) using hexanes as eluent. NMR spectra were recorded at 500 MHz for proton (^1^H) and 126 MHz for proton-decoupled carbon ^13^C{^1^H} using CDCl_3_. The chemical shifts are reported in δ ppm with reference to the signal of tetramethylsilane set to 0 ppm. Infrared spectra (resolution 0.4 cm^–1^) were acquired with an FTIR instrument equipped with an attenuated total reflectance (ATR) accessory and were processed with SpectraGryph.^3^ GC/MS data were obtained with a capillary gas chromatograph interfaced with a quadrupole, triple-axis mass selective detector operating in the electron impact (EI) mode. Melting points are uncorrected. Photolysis was performed in benzene-d6 in a quartz NMR tube using a medium pressure Hg-Xe lamp (equipped with a 280-400 nm dichroic) at ambient temperature.

A Bruker D8 Quest Eco diffractometer equipped with a graphite monochromated Mo Kα radiation (λ= 0.71073 Å) and PHOTON 50™ CMOS (**c**omplementary **m**etal-**o**xide **s**emiconductor) detector was used to collect X-ray diffraction data at 173 K with the Bruker Apex 3 suite of programs.^4^ Frames were integrated with a narrow-frame algorithm using the Bruker data reduction software package SAINT+ ^5^and absorption effects were corrected with the multi-scan method (SADABS).^6^ The Olex2 suite of programs^7^ was used to process data along with the Bruker SHELXTL software package^8, 9^ that was used to perform structure solution by direct methods, and refinement by full-matrix least-squares on F^2^. All nonhydrogen atoms were refined anisotropically with suggested weighting factors and the hydrogens were calculated on a riding model. All cif files were validated with the checkCIF/Platon facility of IUCr that was implemented through Olex2.^7^

**Computational Procedures.**

The quantum chemistry program Orca (version 5.0) was used to perform all calculations.^10^ Geometry optimization calculations for singlet and triplet **4**, **TS**, and **5** were performed using double hybrid density functional theory (B2PLYP)^11^ in combination with Ahlrich’s def2-TZVP^12^ using the auxiliary basis sets def2/J^13^ and def2-TZVP/C.^14, 15^ Geometry optimization calculations for **8** and **9** were done with hybrid density functional theory (B3LYP^16, 17^) using Ahlrich’s def2-SVP^12^ and the auxiliary basis set def2/J.^13^ All single point calculations utilized Domain-based Local Pair Natural Orbital-Coupled Cluster [DLPNO-CCSD(T)] methods.^18-20^ Calculations were universally performed in combination with The Resolution-of-Identity option^21^ and the Chain-of-Spheres^22, 23^ algorithm (RIJCOSX) were employed to accelerate SCF and exchange integral calculations respectively. All calculations used Grimme’s atom pairwise dispersion correction with Becke-Johnson damping (D3BJ).^24, 25^ Frequency calculations were performed to verify the stationary points as minima (0 imaginary frequency) or maxima (1 imaginary frequency). T1 diagnostic^26^ values for all CCSD(T) calculations were ≤0.02, suggesting the species had negligible multireference character and were sufficiently represented by the wavefunctions. ChemCraft^27^ was used to visualize computational data.

**Synthetic Procedures.**

*Synthesis of 2',11a'-diphenyl-1a,9b-dihydrospiro[cyclopropa[l]phenanthrene-1,11'-cyclopropa[1,5]cyclopenta[1,2-l]phenanthren]-1'(11a'H)-one (****8****)*

The dibromo derivative **1** (1.75 g, 5.00 mmol) was dissolved in THF (30 mL) in a 100 mL three-necked flask under argon with a magnetic stir bar. The solution was cooled to -74℃, and a slight excess of *n*-BuLi (3.4 mL, 1.6 M in hexanes, 5.44 mmol) was added to the solution, which adopted a green hue. The reaction was allowed to stir at low temperature for 20 minutes, and phencyclone (**7**, 1.83 g, 4.78 mmol) in THF (30 mL) was added to the solution slowly over 10 minutes, and the solution turned a reddish-brown color upon addition. The solution was kept at -70℃ for two hours, and it was allowed to warm to room temperature, where it stirred for the next 14 hours. The reaction was then quenched by addition of H_2_O (30 mL), the aqueous layer was extracted with CH_2_Cl_2_ (3 × 30 mL), and the organic layer was washed with brine (3 × 30 mL) and dried with sodium sulfate. **8** was isolated as a yellow solid using flash-column chromatography (0:100 🡪 10:90 ethyl acetate:hexanes). The final yield was 1.566 g (56%); mp: decomposes at 153℃ into an orange solid, then again at 188℃ into a red solid, and finally into a black oil at 251℃. ^1^H NMR (500 MHz, CDCl_3_): δ 7.80 – 7.53 (m, 3H), 7.49 (dd, *J* = 8.0, 1.1 Hz, 1H), 7.44 – 7.35 (m, 3H), 7.35 – 7.22 (m, 6H), 7.19 (td, *J* = 7.6, 1.4 Hz, 1H), 7.15 – 7.02 (m, 2H), 6.99 – 6.91 (m, 2H), 6.87 (td, *J* = 7.7, 1.4 Hz, 1H), 6.81 – 6.70 (m, 3H), 6.67 (td, *J* = 7.6, 1.1 Hz, 1H), 6.57 (td, *J* = 7.6, 1.2 Hz, 1H), 6.42 (dd, *J* = 8.0, 1.4 Hz, 1H), 6.12 (dd, *J* = 7.9, 1.3 Hz, 1H), 3.75 (d, *J* = 7.9 Hz, 1H), 3.32 (d, *J* = 8.0 Hz, 1H). ^13^C NMR (126 MHz, CDCl_3_): δ 201.4, 163.0, 134.2, 133.2, 132.9, 132.6, 132.5, 131.1, 130.5, 130.0, 129.6, 129.3, 129.2 (2 carbon resonances), 129.1, 128.9, 128.7, 128.2, 128.1, 127.9, 127.8, 126.9 (2 carbon resonances), 126.8 (2 carbon resonances), 126.7, 126.6, 126.1, 126.0, 123.3, 123.1, 122.6, 122.1, 64.9, 50.4, 41.3, 27.1, 23.3. FTIR: ν 3061.3, 3030.9, 1690.5, 1594.3, 1481, 1444.8 cm^-1^. HRMS (ESI): [M+H]^+^ calcd for C_44_H_29_O: 573.2213; found: 573.2231.

*Synthesis of 9,11-diphenyl-8b,18b-dihydrotetrabenzo[a,c,fg,op]tetracen-10-ol (****10****)*

Several approaches to phenol **10** are delineated below. Methods A and B afforded the white form of the solid, while Methods C and D yielded the red form. Both the red and white **10** had identical NMR and IR spectra; upon heating, red **10** decomposed into a viscous black oil at 251℃, and white **10** began decomposing into a black solid at 280℃. ^1^H NMR (500 MHz, CDCl_3_): δ 8.47 (dd, *J* = 8.4, 1.4 Hz, 1H), 8.32 – 8.24 (m, 1H), 7.83 (dd, *J* = 7.6, 1.3 Hz, 1H), 7.79 (ddt, *J* = 7.7, 2.0, 1.0 Hz, 1H), 7.69 – 7.63 (m, 2H), 7.60 (dd, *J* = 7.8, 1.3 Hz, 1H), 7.55 – 7.39 (m, 9H), 7.36 – 7.26 (m, 4H), 7.06 (dddd, *J* = 12.8, 8.5, 6.7, 1.2 Hz, 2H), 6.90 (td, *J* = 7.5, 1.3 Hz, 1H), 6.85 (dt, *J* = 7.4, 1.2 Hz, 1H), 6.57 (dt, *J* = 7.8, 1.3 Hz, 1H), 5.36 (s, 1H), 4.55 (d, *J* = 5.4 Hz, 1H), 4.51 (d, *J* = 5.4 Hz, 1H). ^13^C NMR (126 MHz, CDCl_3_): δ 149.9, 138.9, 137.2, 137.0, 135.5, 135.2, 134.7, 134.3, 134.2, 131.6, 131.3, 130.5, 130.3 (2 carbon resonances), 130.2, 129.8, 129.7, 129.4, 129.2 (2 carbon resonances) 128.9 (3 carbon resonances) 128.1 (3 carbon resonances), 128.0, 127.7, 127.5, 127.4, 127.2, 126.8, 126.6, 126.2, 125.8, 125.2, 124.6, 123.4, 123.2, 122.8, 122.2, 121.0, 43.5, 40.4. FTIR: ν 3514.9, 3060.9, 3028.8, 2952, 2924.2, 2862.9, 1600.6, 1577.4, 1482.1, 1442.3 cm^-1^. HRMS (ESI): [M+H]^+^ calcd for C_44_H_29_O: 573.2213; found: 573.2228.

1. *Photochemical rearrangement of* ***8***: 21.3 mg of **8** was dissolved in C_6_D_6_ (1 mL) in a quartz NMR tube. Photolysis over the course of 3 hours resulted in the near complete conversion (97%) of **8** into the phenol **10**, as determined by NMR. Subsequent purification of the photolysate with silica-gel flash-column chromatography (0:100 🡪 20:80 ethyl acetate:hexanes) afforded pure **10** as a white solid. Isolated yield: 19.7 mg (92%).
2. *Thermochemical rearrangement of* ***8*** *in chloroform*: 23.0 mg of **8** was dissolved in CHCl_3_ (3 mL) in a 15 mL pressure tube with stirring. Heating to 100℃ for 1 hour resulted in near complete conversion (95%) of **8** into the phenol **10**, as determined by NMR. Subsequent purification of the off-white solid with silica-gel flash-column chromatography (0:100 🡪 20:80 ethyl acetate:hexanes) afforded pure **10** as a white solid. Isolated yield: 20.1 mg (87%).
3. *Thermochemical rearrangement of* ***8*** *in hexanes*: 27.3 mg of **8** was suspended in hexanes (3 mL) in a 15 mL pressure tube with stirring. Heating to 100℃ for 1 hour resulted in the formation of a brick red solid. Vacuum filtration was used to isolate the **10** as a red powder (10.4 mg; 38%), though much of the solid was left behind due to the difficulty of removing it from the pressure tube. The remaining red solid was dissolved in CH_2_Cl_2_ and combined with the filtrate. Removal of solvent and purification with silica-gel flash-column chromatography (0:100 🡪 20:80 ethyl acetate:hexanes) afforded **10** (12.2 mg; 45%) as a white solid.
4. *Trace amounts from reaction to make spiropentane (****8****):* In several of the column fractions containing spiropentane **8**, trace amounts of phenol **10** were found, readily observable because of their tendency to grow as bright red crystals.


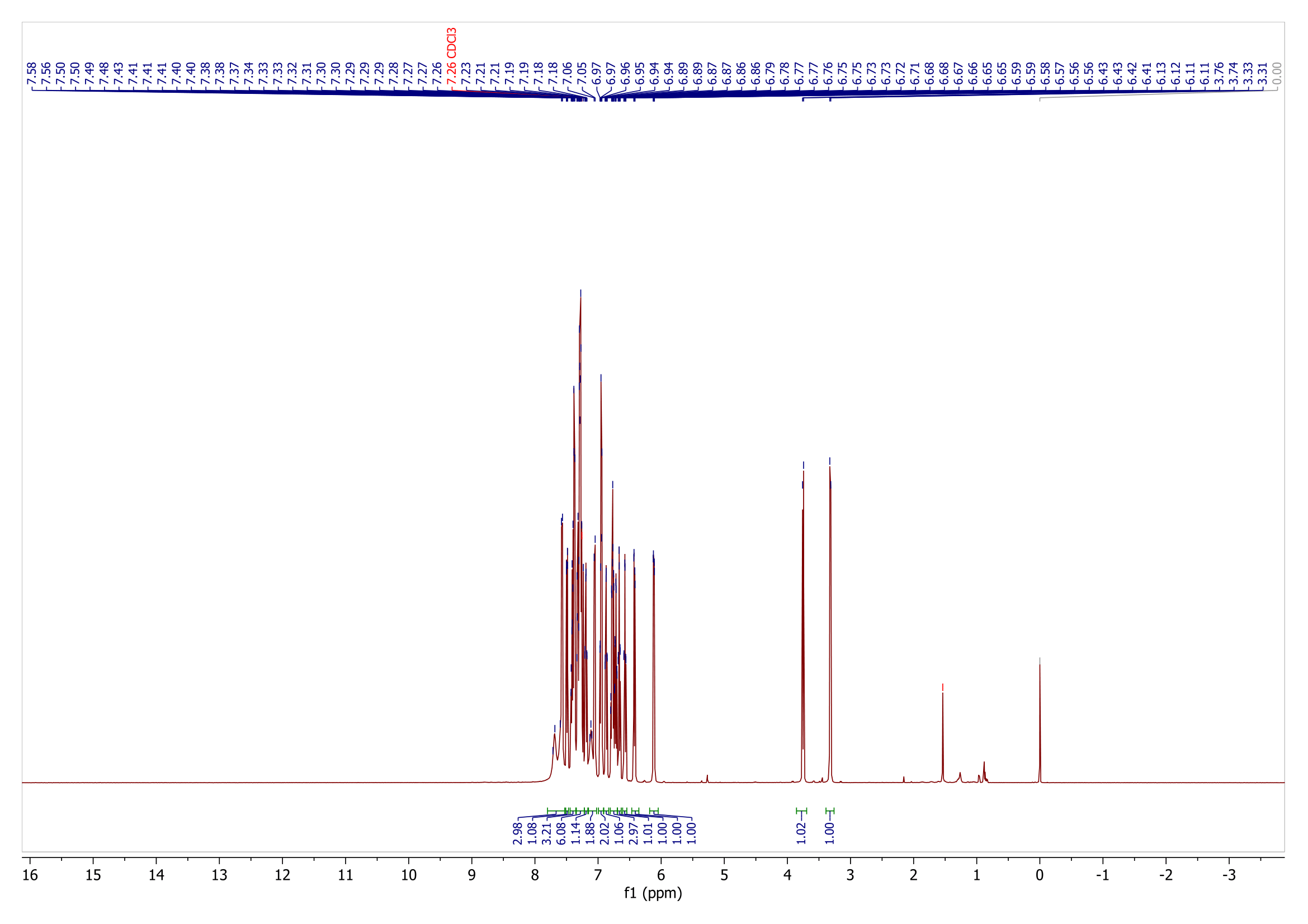

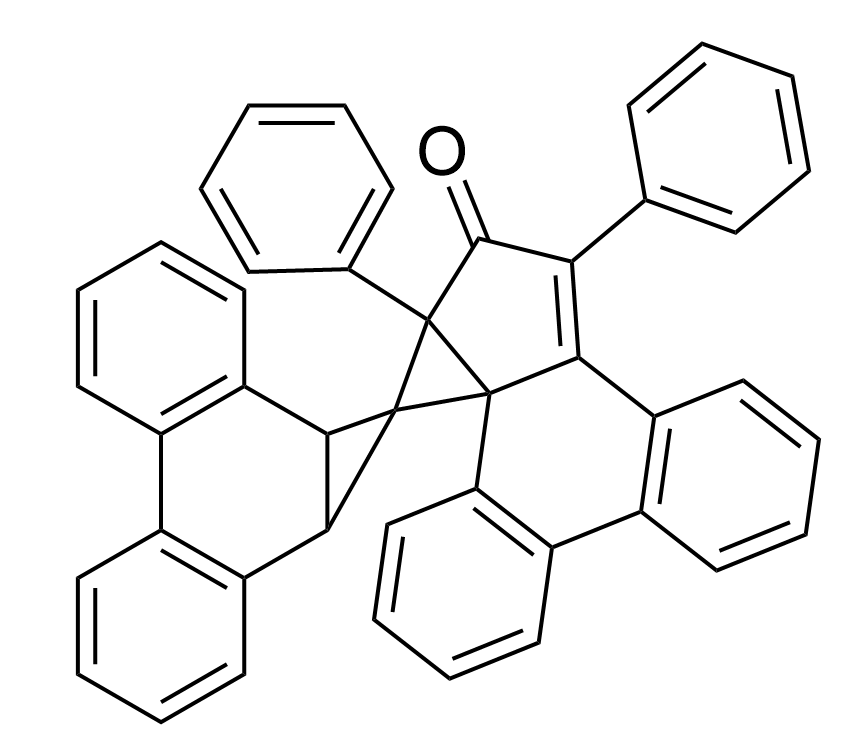


^1^H, 500MHz, CDCl_3_

**8**


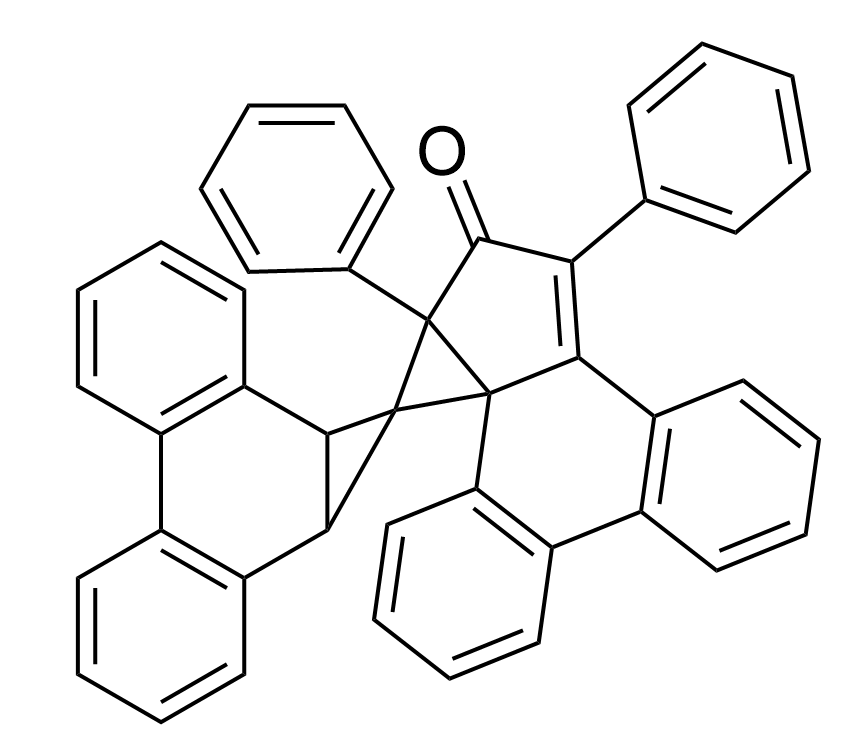

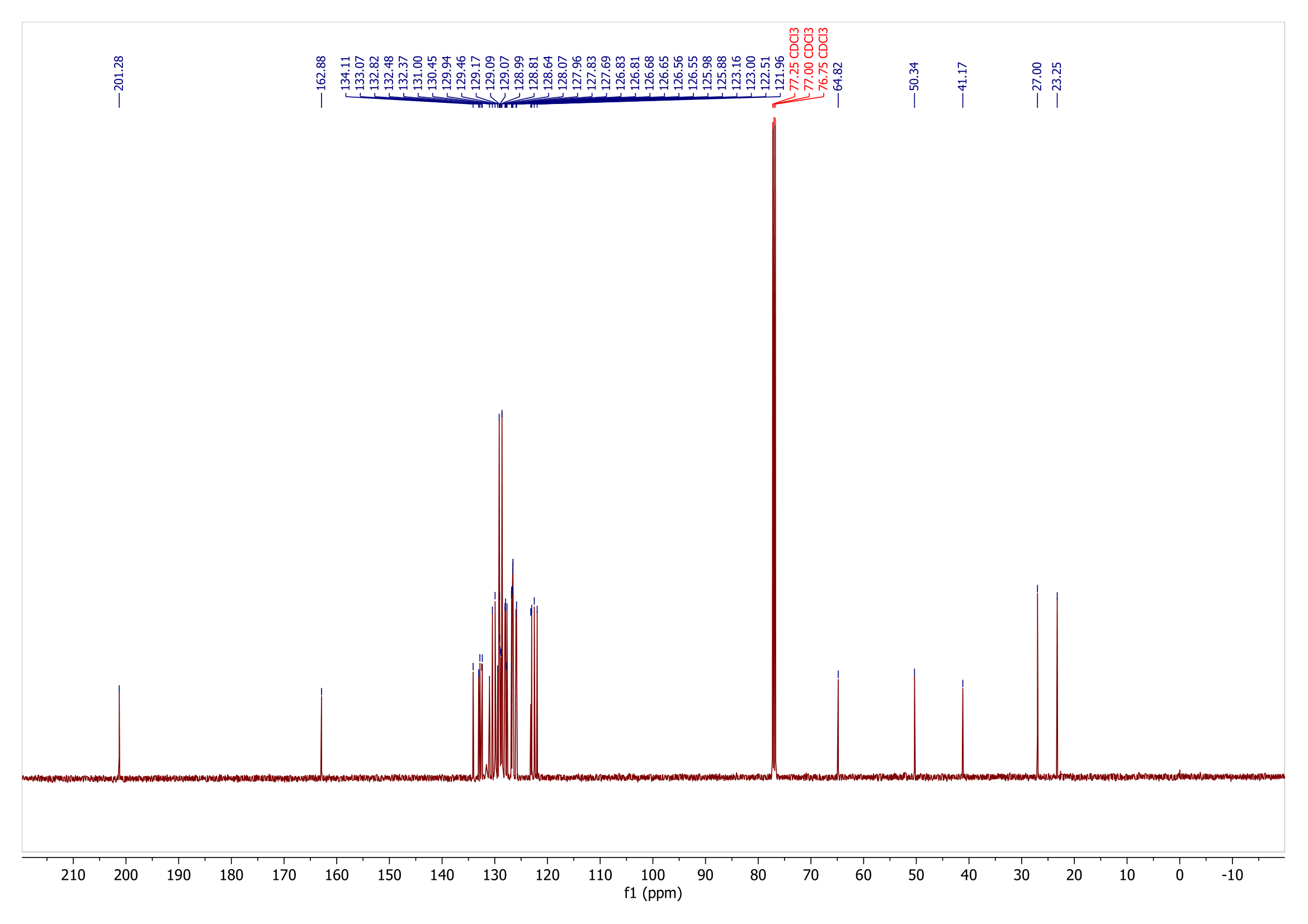


**8**

^13^C{^1^H}, 126 MHz, CDCl_3_


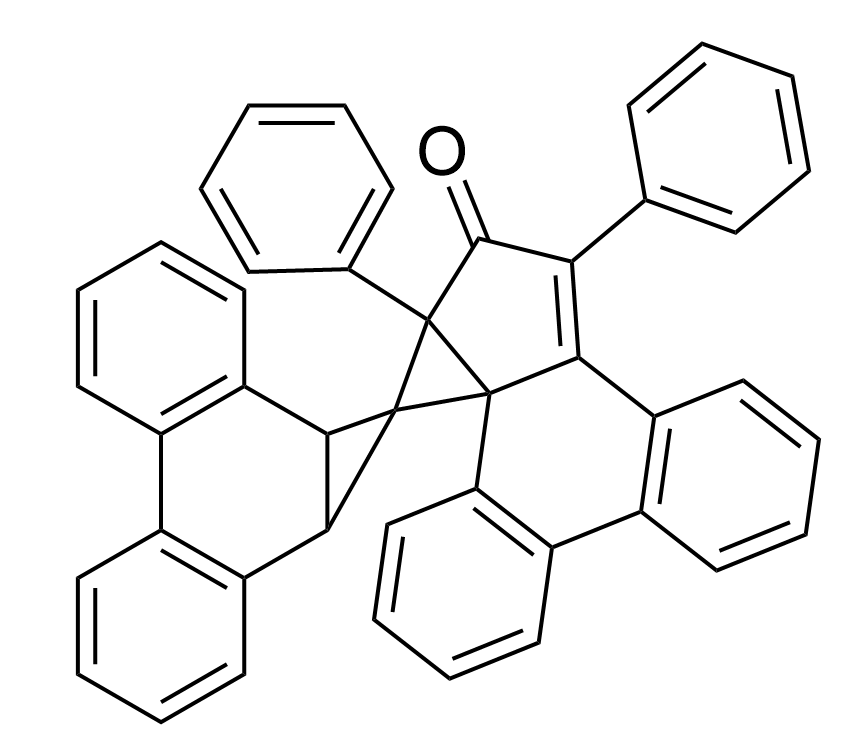


**8**


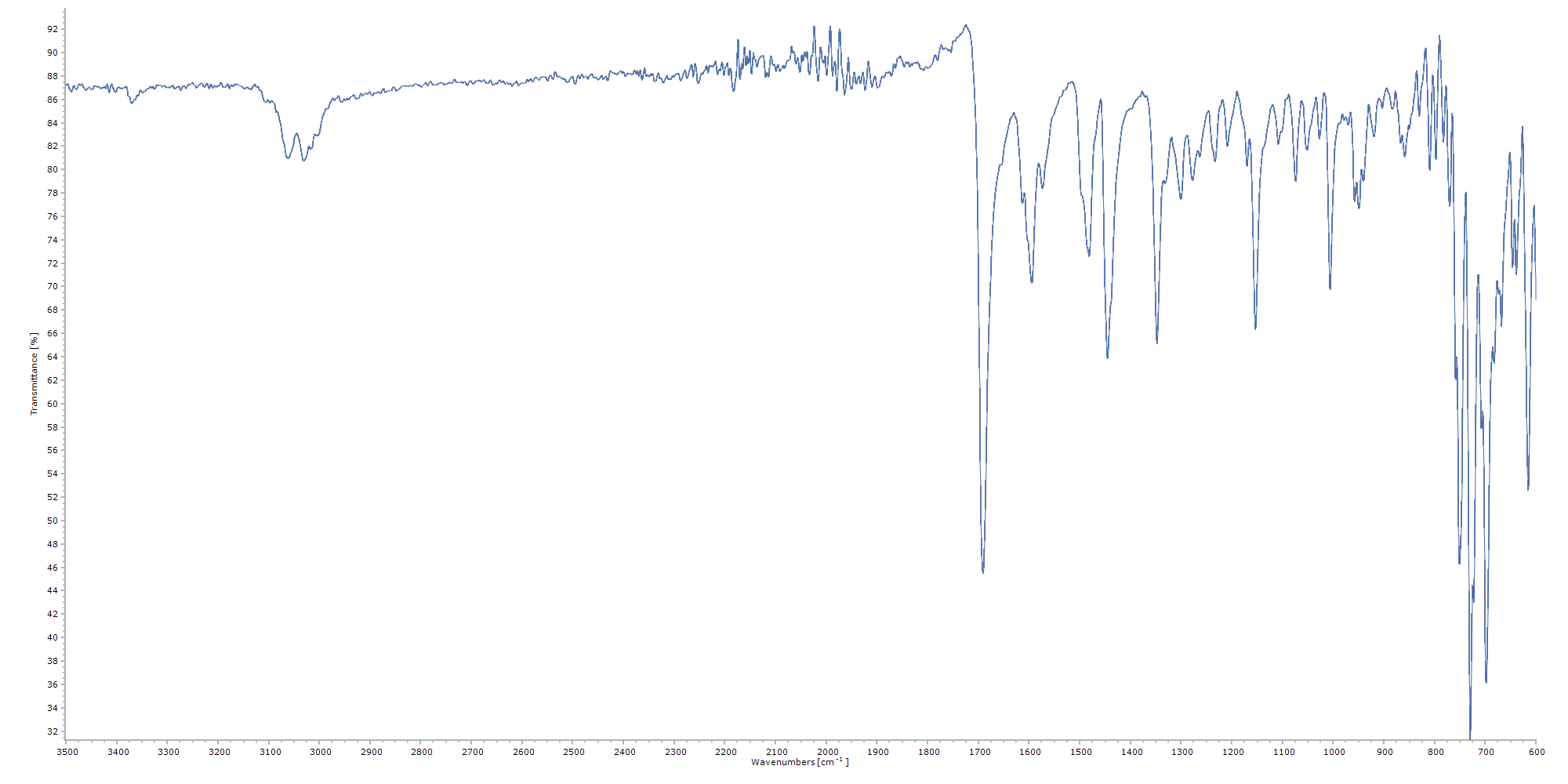

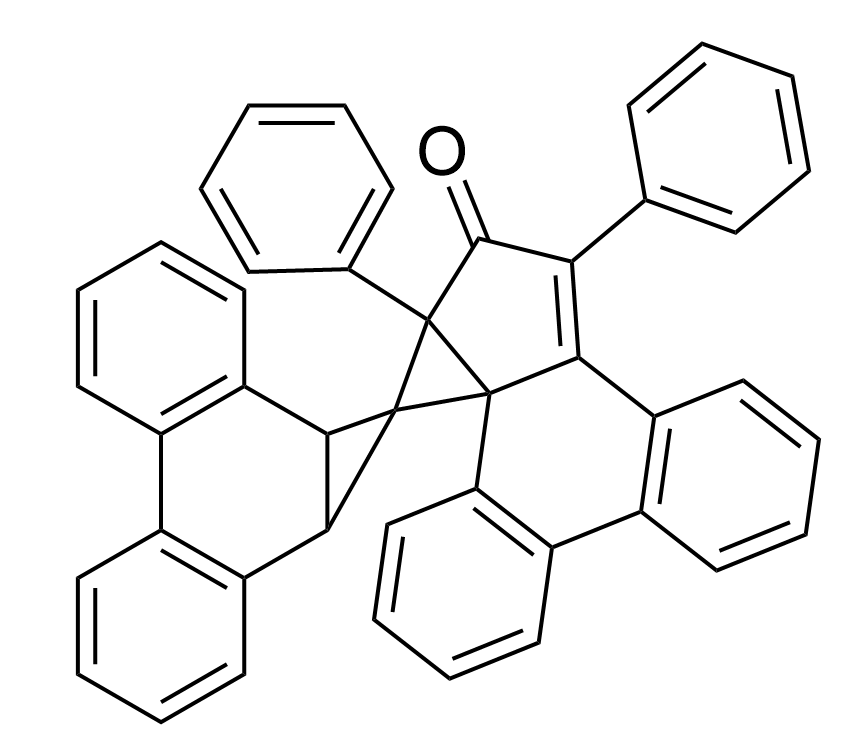


**8**


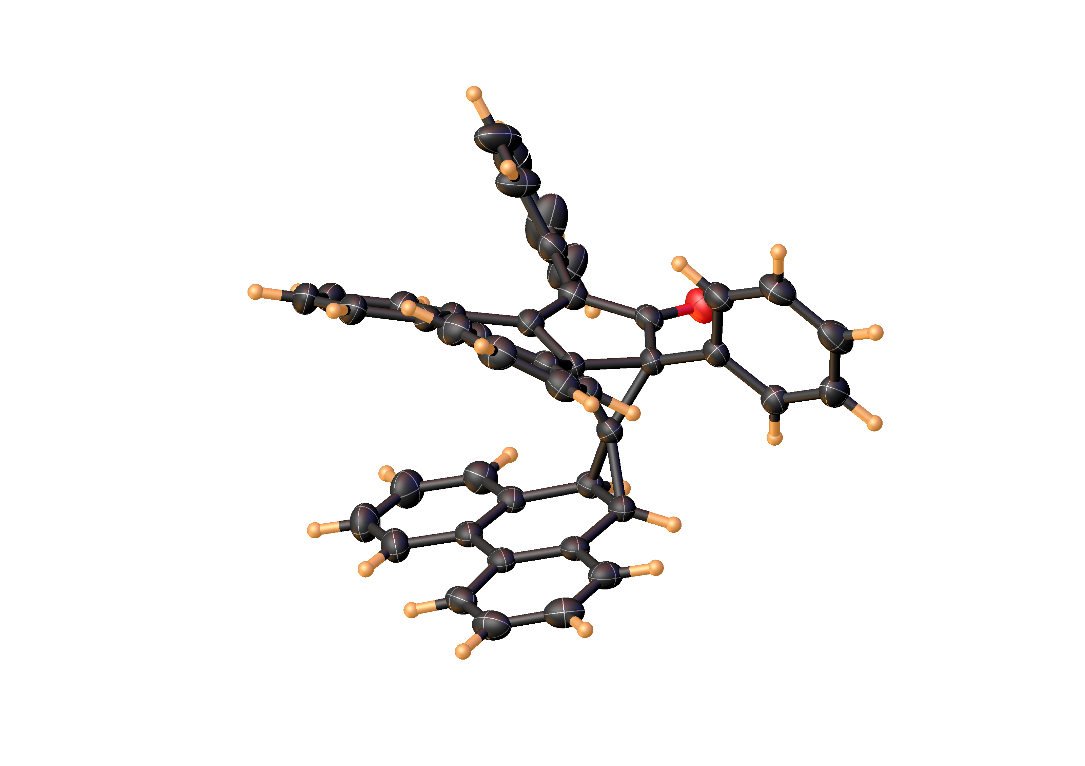


| Empirical formula | C_44_H_28_O |
| --- | --- |
| Formula weight | 572.66 |
| Temperature/K | 173.00 |
| Crystal system | Triclinic |
| Space group | *P*-1 |
| a/Å | 10.8608(2) |
| b/Å | 11.9652(3) |
| c/Å | 12.3289(3) |
| α/° | 97.2430(10) |
| β/° | 91.4890(10) |
| γ/° | 110.4780(10) |
| Volume/Å^3^ | 1484.70(6) |
| Z | 2 |
| ρ_calc_g/cm^3^ | 1.281 |
| μ/mm^‑1^ | 0.075 |
| F(000) | 600.0 |
| Crystal size/mm^3^ | 0.232 × 0.211 × 0.149 |
| Radiation | Mo Kα (λ = 0.71073) |
| 2Θ range for data collection/° | 4.014 to 54.974 |
| Index ranges | -14 ≤ h ≤ 14, -15 ≤ k ≤ 15, -16 ≤ l ≤ 15 |
| Reflections collected | 32889 |
| Independent reflections | 6792 [Rint = 0.0300, Rsigma = 0.0254] |
| Data/restraints/parameters | 6792/0/406 |
| Goodness-of-fit on F^2^ | 1.052 |
| Final R indexes [I>=2σ (I)] | R1 = 0.0553, wR2 = 0.1154 |
| Final R indexes [all data] | R1 = 0.0832, wR2 = 0.1354 |
| Largest diff. peak/hole / e Å^-3^ | 0.30/-0.21 |
| CCDC Number | 2261485 |


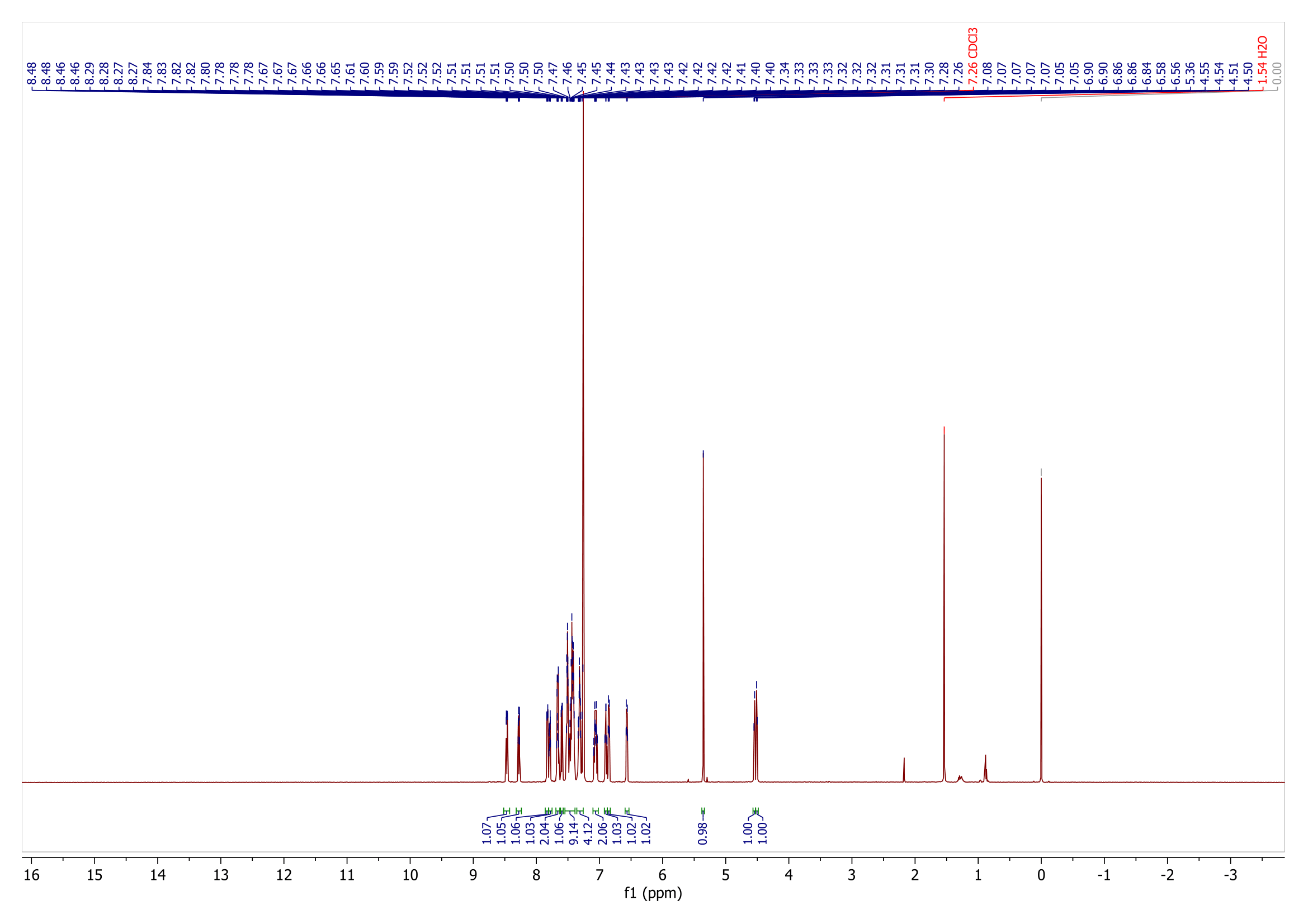


^1^H, 500MHz, CDCl_3_


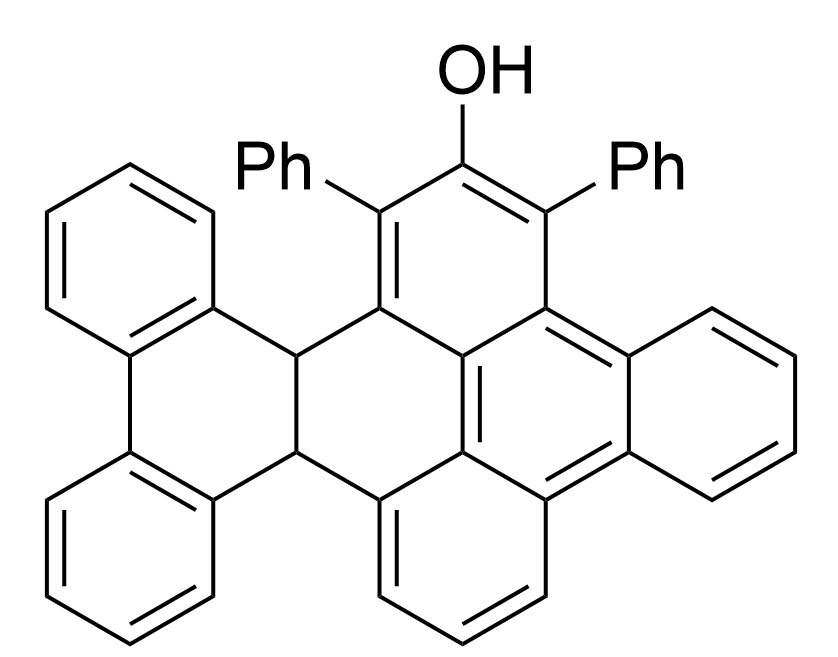


**10**


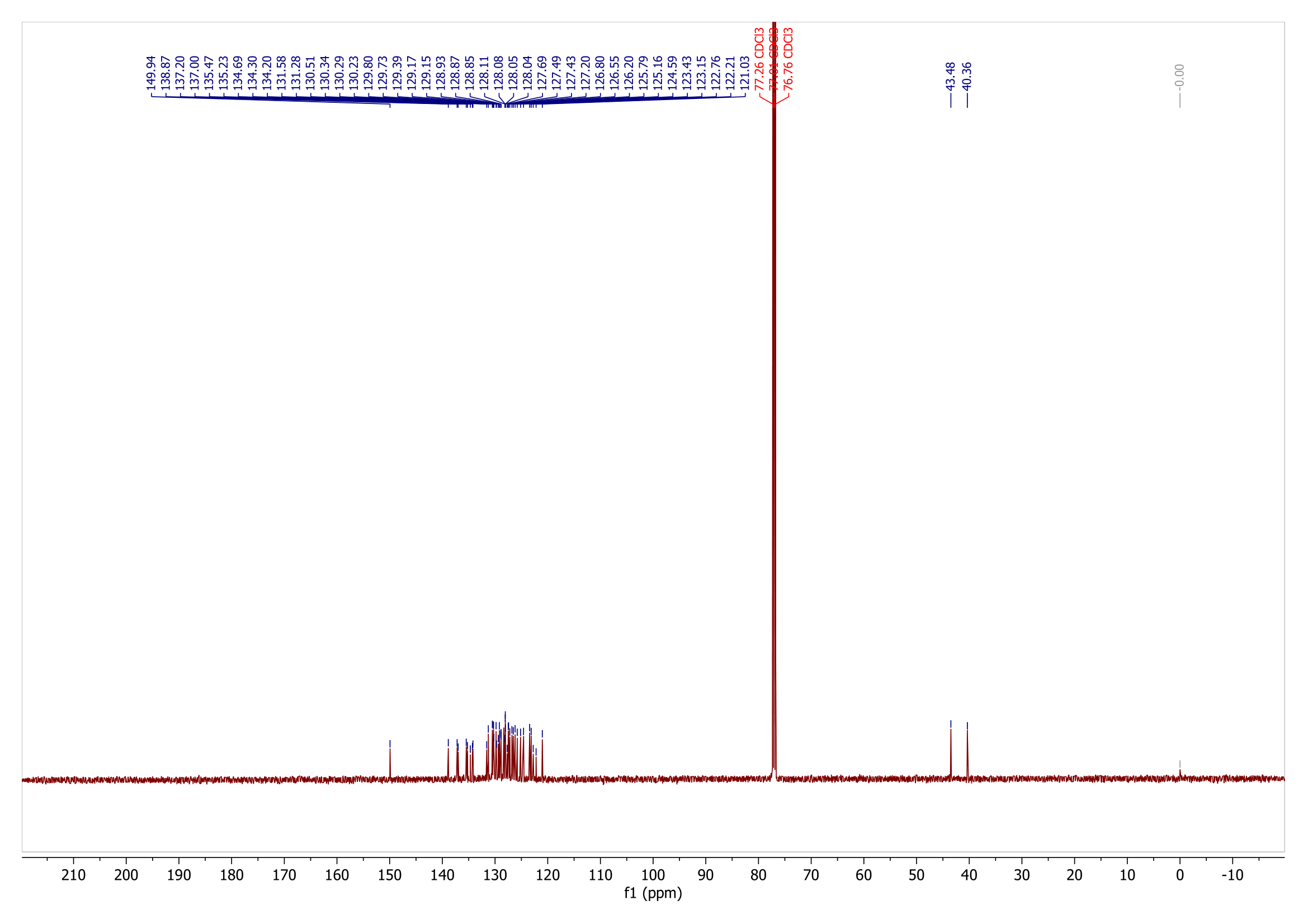


^13^C{^1^H}, 126 MHz, CDCl_3_


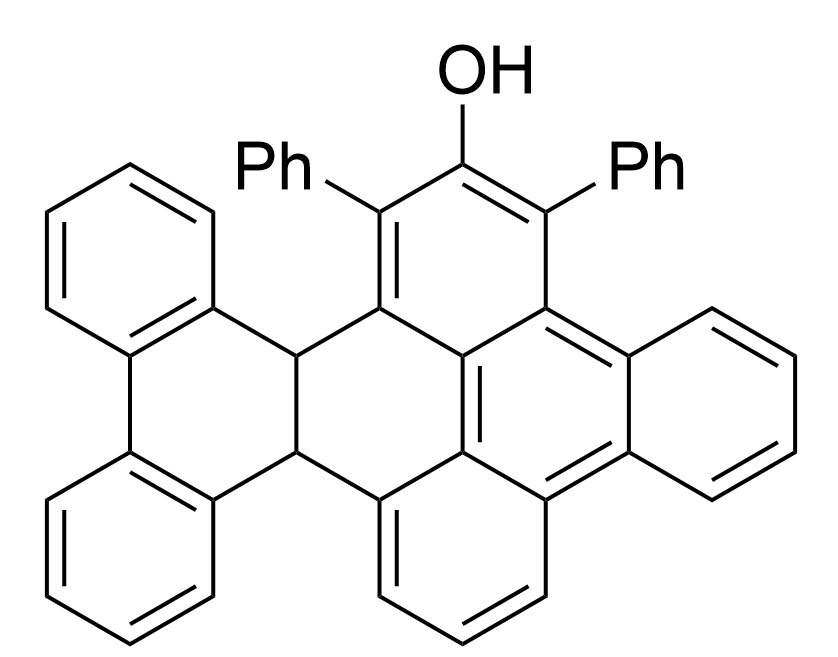


**10**


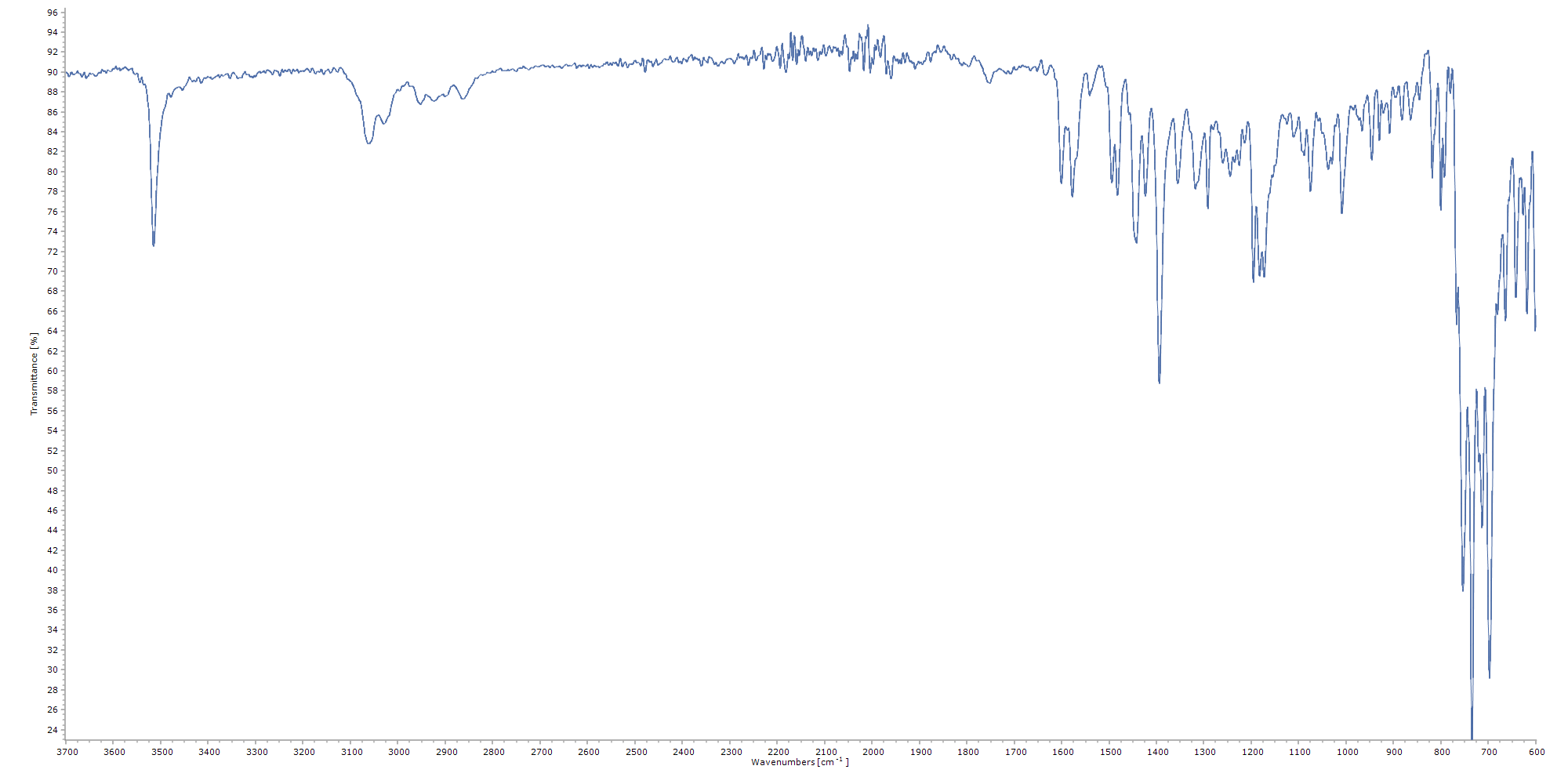


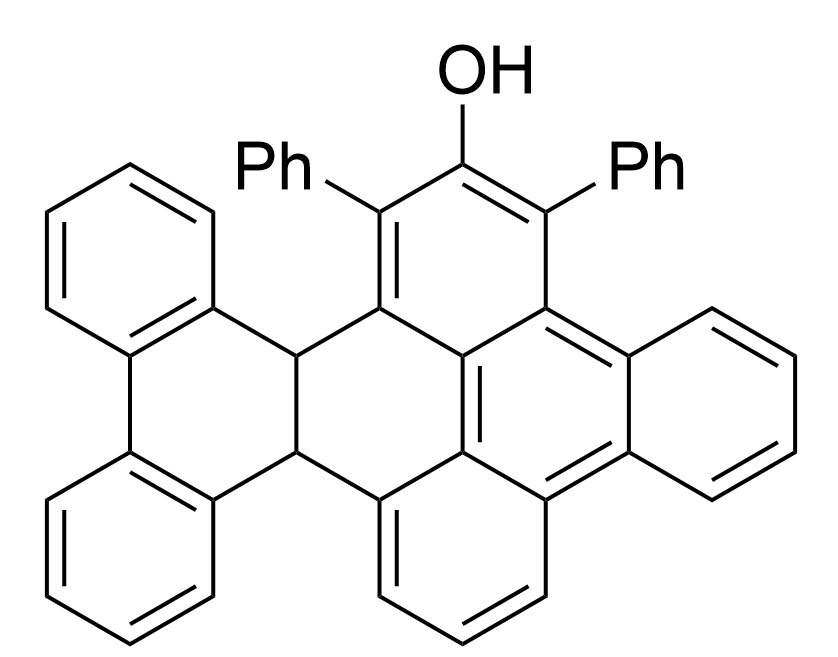


**10**


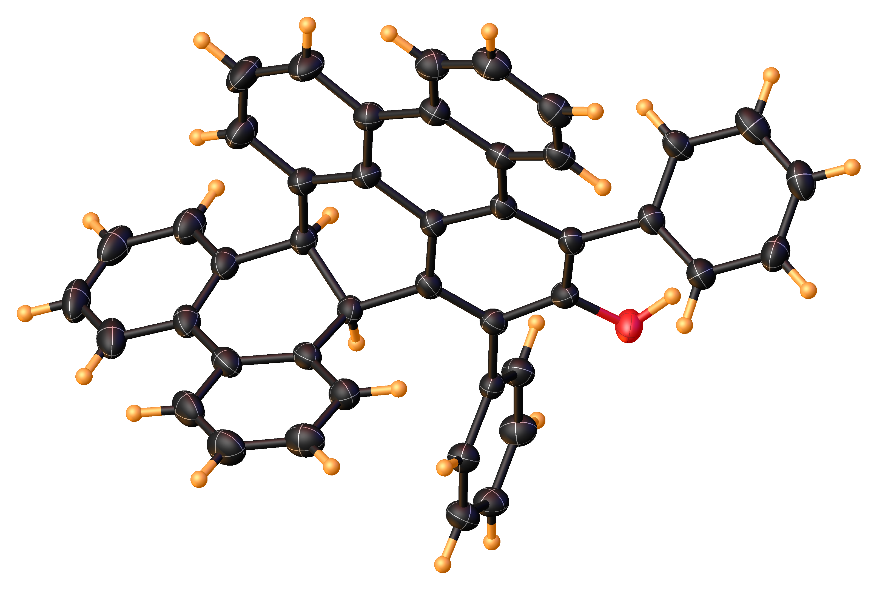


| Empirical formula | C_44_H_28_O |
| --- | --- |
| Formula weight | 572.71 |
| Temperature/K | 173.00 |
| Crystal system | Triclinic |
| Space group | *P*-1 |
| a/Å | 6.2756(2) |
| b/Å | 10.5246(4) |
| c/Å | 22.7127(8) |
| α/° | 76.789(2) |
| β/° | 89.910(2) |
| γ/° | 78.481(2) |
| Volume/Å^3^ | 1429.49(9) |
| Z | 2 |
| ρcalcg/cm^3^ | 1.331 |
| μ/mm^‑1^ | 0.078 |
| F(000) | 600.3 |
| Crystal size/mm^3^ | 0.296 × 0.086 × 0.083 |
| Radiation | Mo Kα (λ = 0.71073) |
| 2Θ range for data collection/° | 4.06 to 55.24 |
| Index ranges | -8 ≤ h ≤ 8, -13 ≤ k ≤ 13, -29 ≤ l ≤ 29 |
| Reflections collected | 32645 |
| Independent reflections | 6605 [Rint = 0.0597, Rsigma = 0.0562] |
| Data/restraints/parameters | 6605/0/410 |
| Goodness-of-fit on F^2^ | 1.152 |
| Final R indexes [I>=2σ (I)] | R1 = 0.0768, wR2 = 0.1707 |
| Final R indexes [all data] | R1 = 0.1335, wR2 = 0.2087 |
| Largest diff. peak/hole / e Å^-3^ | 0.54/-0.49 |
| CCDC Number | 2261429 |

**
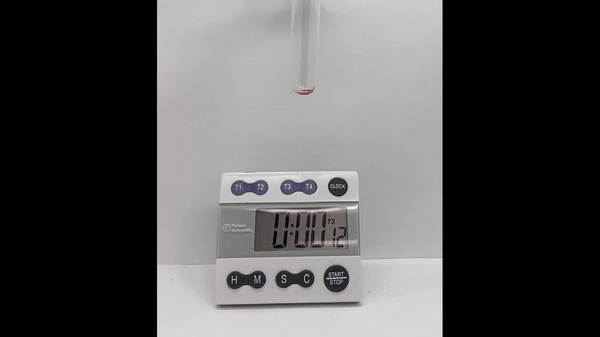
**

**Computational Data**

*Optimized energies, coordinates, frequencies, and geometry for Singlet* ***4****; B2PLYP/def2-TZVP*

------------

INNER ENERGY

------------

The inner energy is: U= E(el) + E(ZPE) + E(vib) + E(rot) + E(trans)

E(el) - is the total energy from the electronic structure calculation

= E(kin-el) + E(nuc-el) + E(el-el) + E(nuc-nuc)

E(ZPE) - the the zero temperature vibrational energy from the frequency calculation

E(vib) - the the finite temperature correction to E(ZPE) due to population

of excited vibrational states

E(rot) - is the rotational thermal energy

E(trans)- is the translational thermal energy

Summary of contributions to the inner energy U:

Electronic energy ... -577.17976372 Eh

Zero point energy ... 0.19612199 Eh 123.07 kcal/mol

Thermal vibrational correction ... 0.00784742 Eh 4.92 kcal/mol

Thermal rotational correction ... 0.00141627 Eh 0.89 kcal/mol

Thermal translational correction ... 0.00141627 Eh 0.89 kcal/mol

-----------------------------------------------------------------------

Total thermal energy -576.97296177 Eh

Summary of corrections to the electronic energy:

(perhaps to be used in another calculation)

Total thermal correction 0.01067996 Eh 6.70 kcal/mol

Non-thermal (ZPE) correction 0.19612199 Eh 123.07 kcal/mol

-----------------------------------------------------------------------

Total correction 0.20680195 Eh 129.77 kcal/mol

---------------------------------

CARTESIAN COORDINATES (ANGSTROEM)

---------------------------------

C -2.063575 -0.119176 -0.151308

C -0.900923 0.643469 -0.103284

C 0.330153 0.009716 0.000554

C 0.407703 -1.378744 0.079980

C -0.759335 -2.158914 0.038134

C -1.989105 -1.504062 -0.090830

H -3.027459 0.362171 -0.246117

H -0.952538 1.722880 -0.149550

H 1.241520 0.593656 0.031439

H -2.902123 -2.076427 -0.167614

C 1.706724 -2.062074 0.191598

C -0.646145 -3.630098 0.067225

C 0.597159 -4.263936 -0.141125

C 1.866503 -3.488693 -0.216152

C -1.776356 -4.447066 0.234858

C -1.690273 -5.825726 0.170228

C -0.459507 -6.441554 -0.065289

C 0.672999 -5.661596 -0.198909

H -2.736500 -3.994586 0.434637

H -2.579915 -6.425419 0.308190

H -0.387719 -7.519268 -0.119029

H 1.640132 -6.124870 -0.346907

C 2.075775 -3.102823 1.215097

H 2.583478 -1.427315 0.063923

H 2.663261 -3.876550 -0.837751

No imaginary frequency.


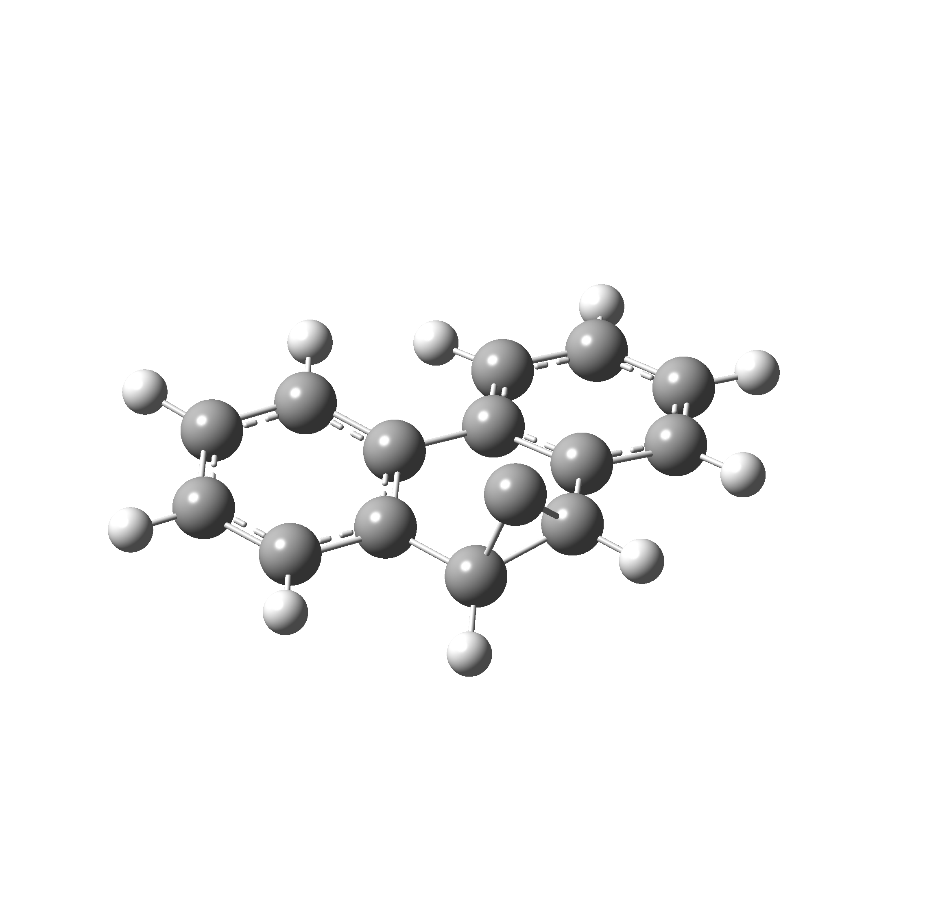


*Optimized energies, coordinates, frequencies, and geometry for Triplet* ***4****; B2PLYP/def2-TZVP*

------------

INNER ENERGY

------------

The inner energy is: U= E(el) + E(ZPE) + E(vib) + E(rot) + E(trans)

E(el) - is the total energy from the electronic structure calculation

= E(kin-el) + E(nuc-el) + E(el-el) + E(nuc-nuc)

E(ZPE) - the the zero temperature vibrational energy from the frequency calculation

E(vib) - the the finite temperature correction to E(ZPE) due to population

of excited vibrational states

E(rot) - is the rotational thermal energy

E(trans)- is the translational thermal energy

Summary of contributions to the inner energy U:

Electronic energy ... -577.34266965 Eh

Zero point energy ... 0.19621004 Eh 123.12 kcal/mol

Thermal vibrational correction ... 0.00762286 Eh 4.78 kcal/mol

Thermal rotational correction ... 0.00141627 Eh 0.89 kcal/mol

Thermal translational correction ... 0.00141627 Eh 0.89 kcal/mol

-----------------------------------------------------------------------

Total thermal energy -577.13600421 Eh

Summary of corrections to the electronic energy:

(perhaps to be used in another calculation)

Total thermal correction 0.01045540 Eh 6.56 kcal/mol

Non-thermal (ZPE) correction 0.19621004 Eh 123.12 kcal/mol

-----------------------------------------------------------------------

Total correction 0.20666544 Eh 129.68 kcal/mol

---------------------------------

CARTESIAN COORDINATES (ANGSTROEM)

---------------------------------

C -2.063953 -0.138002 -0.003167

C -0.916895 0.632868 -0.160012

C 0.320430 0.008222 -0.148432

C 0.431556 -1.372274 0.029653

C -0.725343 -2.172142 0.136941

C -1.963856 -1.514901 0.128707

H -3.039510 0.329218 0.000928

H -0.985900 1.705234 -0.282151

H 1.224880 0.594953 -0.251611

H -2.877672 -2.082297 0.216412

C 1.786893 -1.940073 0.154180

C -0.637784 -3.650934 0.134383

C 0.605407 -4.308887 0.029920

C 1.884528 -3.585427 0.154946

C -1.790500 -4.449083 0.117893

C -1.727495 -5.828071 -0.015099

C -0.496902 -6.458722 -0.165585

C 0.658066 -5.692784 -0.148947

H -2.765095 -3.992584 0.198289

H -2.641485 -6.406559 -0.017029

H -0.438585 -7.531730 -0.287628

H 1.625744 -6.168701 -0.249150

C 2.178417 -2.742056 1.284164

H 2.559293 -1.439172 -0.427995

H 2.709695 -3.993098 -0.427612

No imaginary frequency.


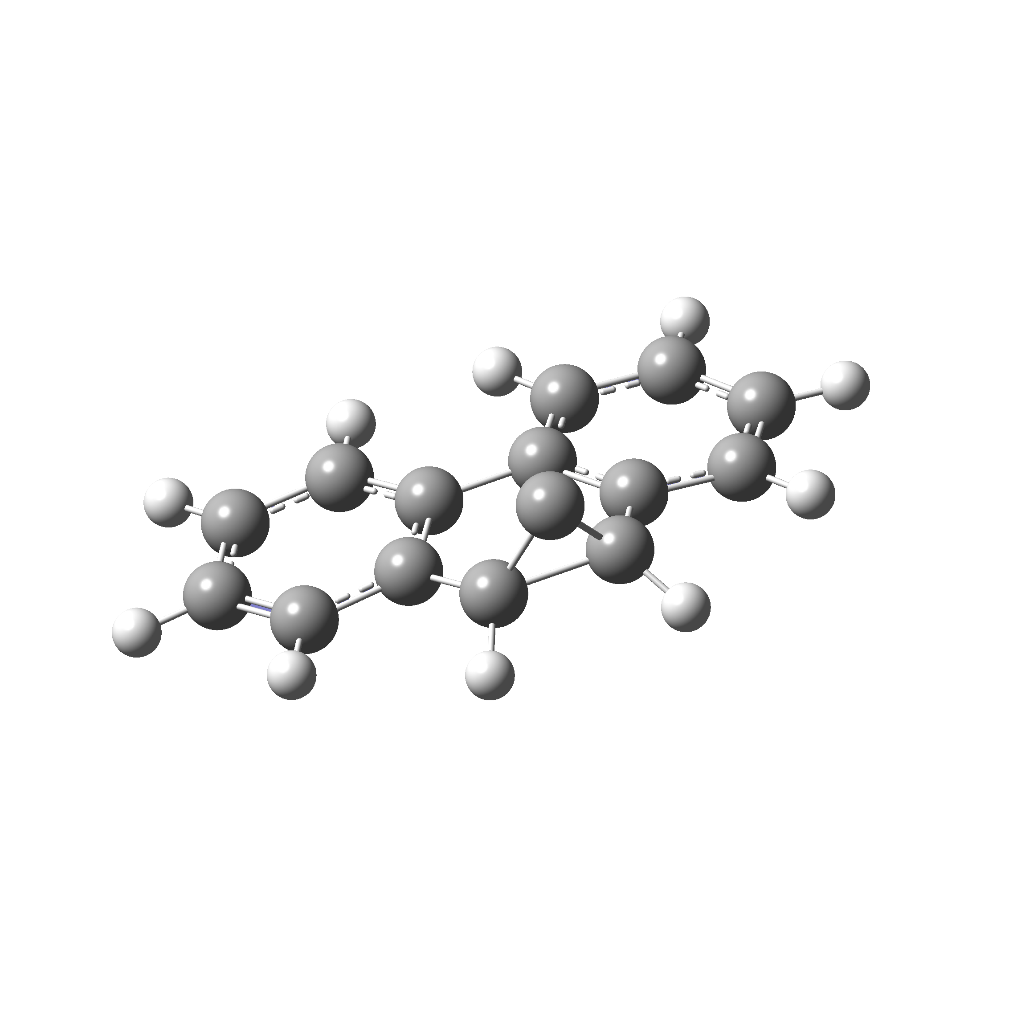


*Optimized energies, coordinates, frequency, and geometry for Allene* ***5****; B2PLYP/def2-TZVP*

------------

INNER ENERGY

------------

The inner energy is: U= E(el) + E(ZPE) + E(vib) + E(rot) + E(trans)

E(el) - is the total energy from the electronic structure calculation

= E(kin-el) + E(nuc-el) + E(el-el) + E(nuc-nuc)

E(ZPE) - the the zero temperature vibrational energy from the frequency calculation

E(vib) - the the finite temperature correction to E(ZPE) due to population

of excited vibrational states

E(rot) - is the rotational thermal energy

E(trans)- is the translational thermal energy

Summary of contributions to the inner energy U:

Electronic energy ... -577.26437086 Eh

Zero point energy ... 0.19789356 Eh 124.18 kcal/mol

Thermal vibrational correction ... 0.00763119 Eh 4.79 kcal/mol

Thermal rotational correction ... 0.00141627 Eh 0.89 kcal/mol

Thermal translational correction ... 0.00141627 Eh 0.89 kcal/mol

-----------------------------------------------------------------------

Total thermal energy -577.05601357 Eh

Summary of corrections to the electronic energy:

(perhaps to be used in another calculation)

Total thermal correction 0.01046374 Eh 6.57 kcal/mol

Non-thermal (ZPE) correction 0.19789356 Eh 124.18 kcal/mol

-----------------------------------------------------------------------

Total correction 0.20835729 Eh 130.75 kcal/mol

---------------------------------

CARTESIAN COORDINATES (ANGSTROEM)

---------------------------------

C -2.004896 -0.264340 -0.434744

C -1.109992 0.638481 0.134385

C 0.054846 0.162884 0.712790

C 0.341902 -1.205043 0.742712

C -0.586414 -2.143879 0.217929

C -1.746198 -1.625193 -0.384601

H -2.904726 0.091714 -0.918397

H -1.313643 1.700652 0.112140

H 0.772638 0.851620 1.140031

H -2.448127 -2.309097 -0.841330

C 1.590861 -1.721919 1.327312

C -0.484766 -3.633173 0.312023

C 0.676099 -4.413398 0.063005

C 1.942706 -3.705991 -0.190705

C -1.669626 -4.330463 0.607899

C -1.726804 -5.715316 0.618587

C -0.591970 -6.463947 0.316258

C 0.597832 -5.809205 0.045649

H -2.559112 -3.767743 0.855234

H -2.657062 -6.210415 0.863353

H -0.634184 -7.544838 0.307657

H 1.495593 -6.374821 -0.169313

C 2.132656 -2.685550 0.615979

H 1.949071 -1.386298 2.292679

H 2.583905 -3.967785 -1.023067

No imaginary frequency.


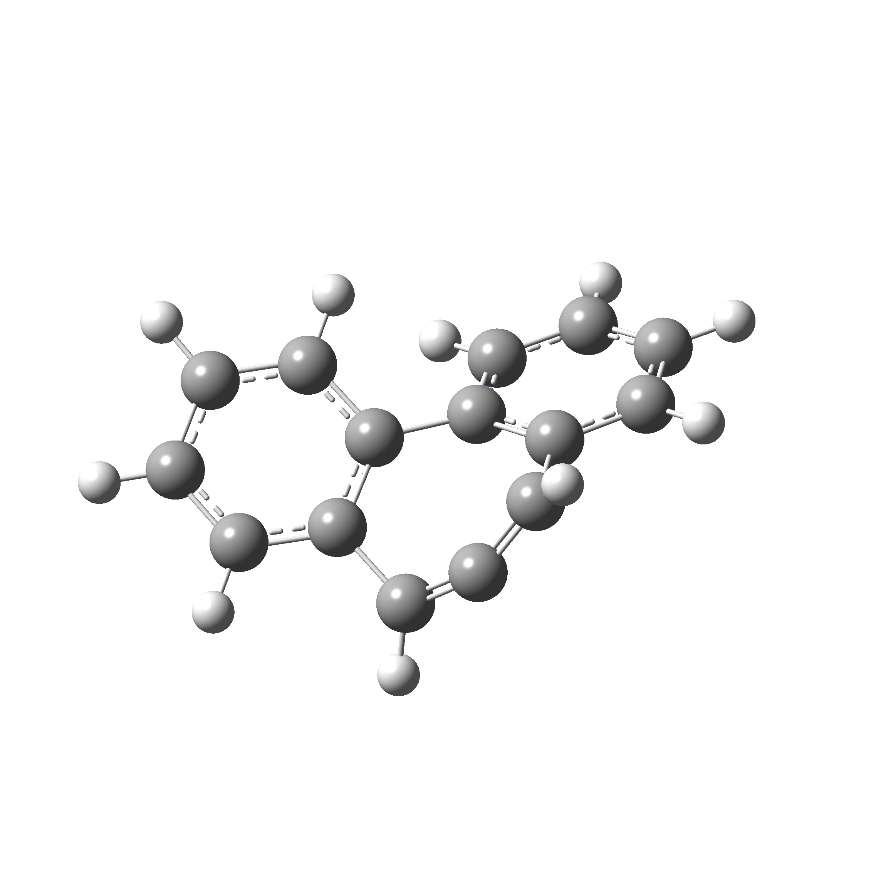


*Optimized energies, coordinates, frequencies, and geometry for transition state* ***TS****;* *B2PLYP/def2-TZVP*

------------

INNER ENERGY

------------

The inner energy is: U= E(el) + E(ZPE) + E(vib) + E(rot) + E(trans)

E(el) - is the total energy from the electronic structure calculation

= E(kin-el) + E(nuc-el) + E(el-el) + E(nuc-nuc)

E(ZPE) - the the zero temperature vibrational energy from the frequency calculation

E(vib) - the the finite temperature correction to E(ZPE) due to population

of excited vibrational states

E(rot) - is the rotational thermal energy

E(trans)- is the translational thermal energy

Summary of contributions to the inner energy U:

Electronic energy ... -577.16765473 Eh

Zero point energy ... 0.19515233 Eh 122.46 kcal/mol

Thermal vibrational correction ... 0.00763618 Eh 4.79 kcal/mol

Thermal rotational correction ... 0.00141627 Eh 0.89 kcal/mol

Thermal translational correction ... 0.00141627 Eh 0.89 kcal/mol

-----------------------------------------------------------------------

Total thermal energy -576.96203367 Eh

Summary of corrections to the electronic energy:

(perhaps to be used in another calculation)

Total thermal correction 0.01046872 Eh 6.57 kcal/mol

Non-thermal (ZPE) correction 0.19515233 Eh 122.46 kcal/mol

-----------------------------------------------------------------------

Total correction 0.20562105 Eh 129.03 kcal/mol

---------------------------------

CARTESIAN COORDINATES (ANGSTROEM)

---------------------------------

C -1.786291 2.743481 0.018132

C -0.631750 3.486491 -0.229717

C 0.594270 2.851191 -0.231871

C 0.706282 1.470020 0.010141

C -0.463709 0.701746 0.175111

C -1.696853 1.377393 0.202404

H -2.752866 3.227807 0.046336

H -0.689964 4.551439 -0.408518

H 1.497605 3.426598 -0.388222

H -2.607957 0.820536 0.360039

C 2.082260 1.006253 0.203683

C -0.381404 -0.753350 0.165124

C 0.871500 -1.382315 0.020793

C 2.177804 -0.760741 0.240875

C -1.530592 -1.563596 0.151386

C -1.459878 -2.929204 -0.045636

C -0.222554 -3.535307 -0.264967

C 0.922947 -2.765203 -0.231944

H -2.503466 -1.114001 0.279229

H -2.366240 -3.519276 -0.050161

H -0.155273 -4.598363 -0.451599

H 1.889423 -3.232760 -0.369360

C 2.718245 0.175946 1.182033

H 2.804588 1.697696 -0.231952

H 2.983874 -1.382482 -0.151339

One imaginary frequency (-368.58 cm^-1^).


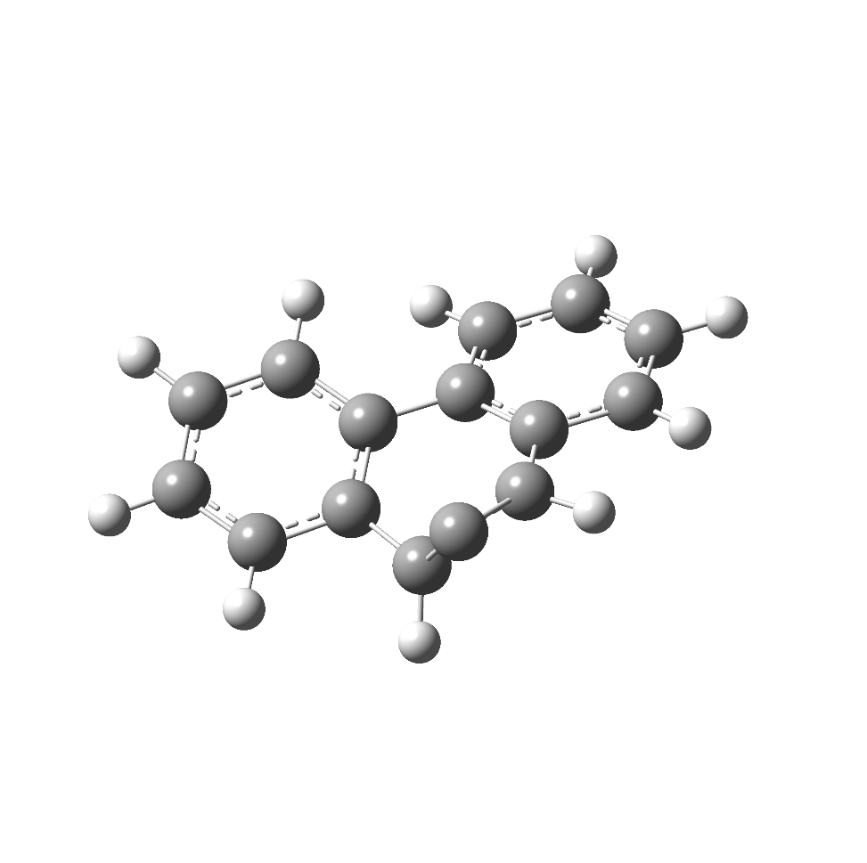


*Single point energies and T1 diagnostics for* ***4*** *to* ***5*** *PES; CCSD(T)/def2-TZVP//B2PLYP/def2-TZVP:*

**4**

------------------------- --------------------

FINAL SINGLE POINT ENERGY -576.351102390249

------------------------- --------------------

T1 diagnostic ... 0.010838638

**5**

------------------------- --------------------

FINAL SINGLE POINT ENERGY -576.431185318525

------------------------- --------------------

T1 diagnostic ... 0.010437101

**TS**

------------------------- --------------------

FINAL SINGLE POINT ENERGY -576.339244921891

------------------------- --------------------

T1 diagnostic ... 0.011373653

*Optimized energies, coordinates, frequencies, and geometry for* ***9****;* *B3LYP/def2-SVP*

------------

INNER ENERGY

------------

The inner energy is: U= E(el) + E(ZPE) + E(vib) + E(rot) + E(trans)

E(el) - is the total energy from the electronic structure calculation

= E(kin-el) + E(nuc-el) + E(el-el) + E(nuc-nuc)

E(ZPE) - the the zero temperature vibrational energy from the frequency calculation

E(vib) - the the finite temperature correction to E(ZPE) due to population

of excited vibrational states

E(rot) - is the rotational thermal energy

E(trans)- is the translational thermal energy

Summary of contributions to the inner energy U:

Electronic energy ... -1766.59217658 Eh

Zero point energy ... 0.58225904 Eh 365.37 kcal/mol

Thermal vibrational correction ... 0.02975966 Eh 18.67 kcal/mol

Thermal rotational correction ... 0.00141627 Eh 0.89 kcal/mol

Thermal translational correction ... 0.00141627 Eh 0.89 kcal/mol

-----------------------------------------------------------------------

Total thermal energy -1765.97732534 Eh

Summary of corrections to the electronic energy:

(perhaps to be used in another calculation)

Total thermal correction 0.03259221 Eh 20.45 kcal/mol

Non-thermal (ZPE) correction 0.58225904 Eh 365.37 kcal/mol

-----------------------------------------------------------------------

Total correction 0.61485124 Eh 385.82 kcal/mol

---------------------------------

CARTESIAN COORDINATES (ANGSTROEM)

---------------------------------

O -3.855777 1.654684 -0.664274

C -2.680957 1.485053 -0.896433

C -2.074766 0.920083 -2.136821

C -2.930927 0.569838 -3.285203

C -4.097032 -0.191313 -3.087873

C -4.919351 -0.519408 -4.168166

C -4.602210 -0.079465 -5.456214

C -3.458560 0.701148 -5.658402

C -2.633530 1.026552 -4.582131

C -0.716896 0.848004 -1.986627

C -0.275942 1.342243 -0.637865

C -1.547415 1.806705 0.084054

C -1.689791 3.065538 0.871333

C -2.358487 3.078966 2.102098

C -2.528655 4.276585 2.801036

C -2.047234 5.476138 2.270282

C -1.403519 5.474822 1.028375

C -1.230059 4.277538 0.332532

C 1.086749 1.916607 -0.519477

C 1.492992 2.621070 0.624632

C 2.771188 3.169324 0.711398

C 3.665392 3.012474 -0.351982

C 3.288619 2.270870 -1.468626

C 2.011626 1.684553 -1.567732

C 1.659727 0.772726 -2.685031

C 0.319031 0.333098 -2.877370

C 0.030633 -0.605586 -3.887980

C 1.027363 -1.087574 -4.727861

C 2.341756 -0.633970 -4.565869

C 2.647686 0.273026 -3.555036

C -0.857483 0.561321 0.511071

C -1.169272 -0.878409 0.702412

C -2.550292 -1.266661 1.077560

C -3.114613 -0.850424 2.306833

C -4.468522 -1.161033 2.542331

C -5.228737 -1.873812 1.618908

C -4.647984 -2.312880 0.425084

C -3.313988 -2.007266 0.165415

C -2.284471 -0.146934 3.318824

C -0.929841 0.171704 3.052514

C -0.326634 -0.104071 1.723576

C -0.147419 0.797830 4.031135

C -0.684444 1.151483 5.267197

C -2.025057 0.860181 5.535845

C -2.803166 0.213524 4.578175

H -4.356126 -0.518769 -2.080709

H -5.818473 -1.118032 -4.000287

H -5.249247 -0.334344 -6.299461

H -3.211840 1.063573 -6.659735

H -1.744959 1.641415 -4.740965

H -2.748610 2.150363 2.512043

H -3.043480 4.268109 3.765034

H -2.179036 6.412466 2.818716

H -1.033318 6.410089 0.600631

H -0.722195 4.275285 -0.634362

H 0.792916 2.752830 1.448908

H 3.063244 3.729101 1.603160

H 4.662003 3.458044 -0.307031

H 4.006670 2.147882 -2.279842

H -0.991523 -0.963411 -4.001158

H 0.784281 -1.819621 -5.501422

H 3.136534 -1.004017 -5.218311

H 3.686640 0.575750 -3.425657

H -0.643473 -1.567943 0.034370

H -4.947834 -0.840044 3.467049

H -6.277981 -2.089672 1.833789

H -5.234490 -2.878526 -0.303009

H -2.851948 -2.321948 -0.773948

H 0.753445 -0.285841 1.724209

H 0.896035 1.029068 3.800568

H -0.065136 1.654650 6.013494

H -2.466263 1.130561 6.498272

H -3.840645 -0.012657 4.823191

No imaginary frequency.


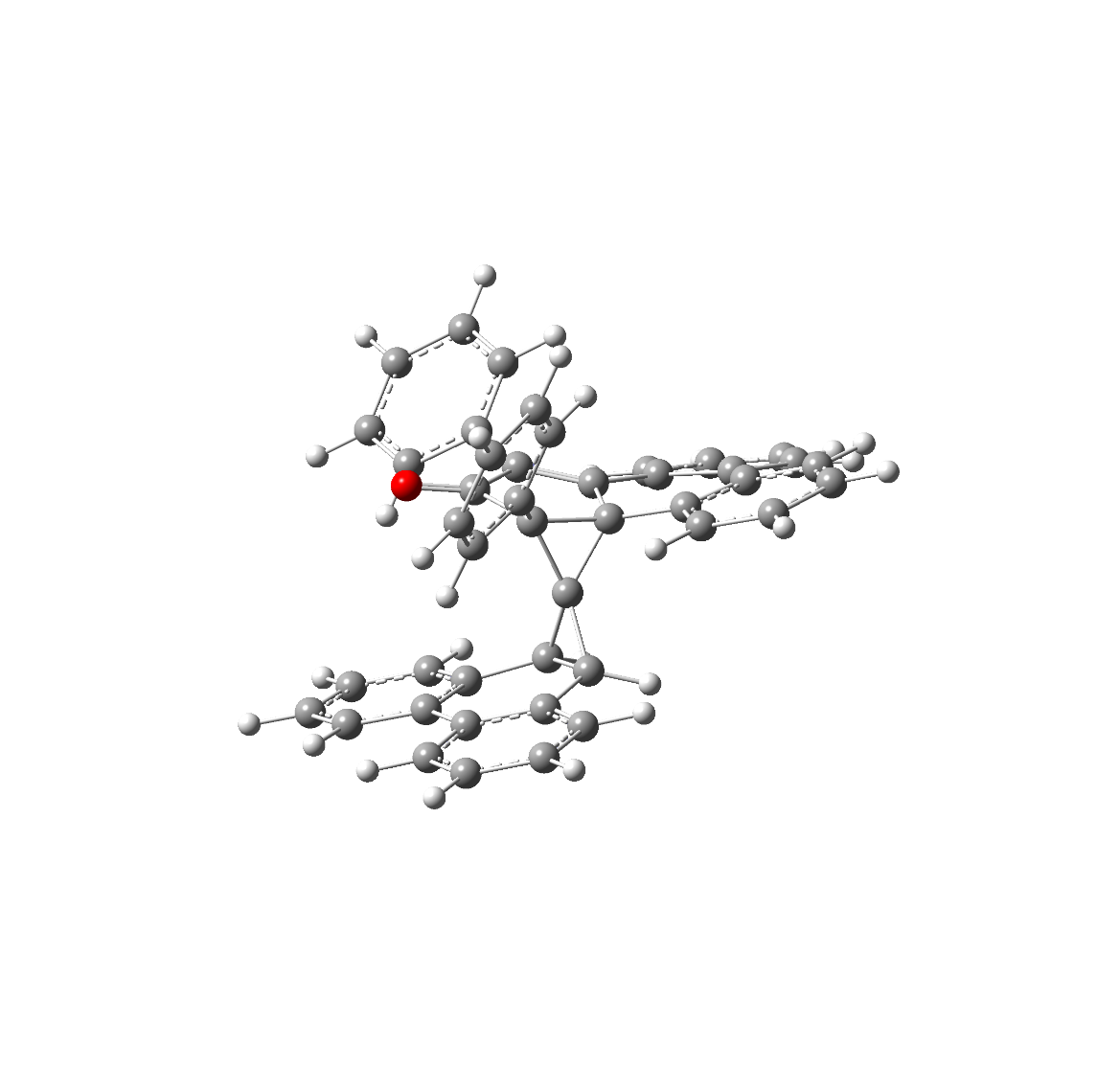


*Optimized energies, coordinates, frequencies, and geometry for* ***8****; B3LYP/def2-SVP*

------------

INNER ENERGY

------------

The inner energy is: U= E(el) + E(ZPE) + E(vib) + E(rot) + E(trans)

E(el) - is the total energy from the electronic structure calculation

= E(kin-el) + E(nuc-el) + E(el-el) + E(nuc-nuc)

E(ZPE) - the the zero temperature vibrational energy from the frequency calculation

E(vib) - the the finite temperature correction to E(ZPE) due to population

of excited vibrational states

E(rot) - is the rotational thermal energy

E(trans)- is the translational thermal energy

Summary of contributions to the inner energy U:

Electronic energy ... -1766.59972300 Eh

Zero point energy ... 0.58204029 Eh 365.23 kcal/mol

Thermal vibrational correction ... 0.02971823 Eh 18.65 kcal/mol

Thermal rotational correction ... 0.00141627 Eh 0.89 kcal/mol

Thermal translational correction ... 0.00141627 Eh 0.89 kcal/mol

-----------------------------------------------------------------------

Total thermal energy -1765.98513194 Eh

Summary of corrections to the electronic energy:

(perhaps to be used in another calculation)

Total thermal correction 0.03255077 Eh 20.43 kcal/mol

Non-thermal (ZPE) correction 0.58204029 Eh 365.23 kcal/mol

-----------------------------------------------------------------------

Total correction 0.61459106 Eh 385.66 kcal/mol

---------------------------------

CARTESIAN COORDINATES (ANGSTROEM)

---------------------------------

O 1.910596 5.054774 10.547504

C 8.141205 8.171610 10.596370

H 8.747174 9.048355 10.356563

C 6.966782 8.308383 11.342482

H 6.641274 9.290674 11.692962

C 6.205220 7.176592 11.624123

H 5.275372 7.269767 12.190958

C 6.593071 5.911834 11.163304

C 5.706118 4.751640 11.409793

H 5.050731 4.826887 12.283497

C 5.136940 4.020594 10.249588

C 4.964736 4.362003 8.793993

C 4.692602 5.820795 8.563953

C 3.531758 6.216068 9.168918

C 2.893289 7.543862 9.211777

C 2.678305 8.285526 8.036179

H 2.994928 7.868196 7.077749

C 2.074809 9.541858 8.086720

H 1.917338 10.105407 7.163449

C 1.665949 10.076566 9.313520

H 1.192529 11.060901 9.353232

C 5.724202 3.635405 7.743074

C 5.714885 2.238210 7.639399

H 5.137716 1.650628 8.350593

C 6.438771 1.588883 6.641334

H 6.413083 0.498635 6.574683

C 7.186427 2.337095 5.728734

H 7.742432 1.840655 4.929758

C 7.228015 3.723995 5.843929

H 7.817336 4.293692 5.124758

C 6.523056 4.398572 6.858029

C 6.647319 5.863384 7.043557

C 7.692854 6.584505 6.439609

H 8.432207 6.058709 5.835260

C 7.828368 7.959136 6.617730

H 8.655913 8.487951 6.138083

C 6.920480 8.653744 7.423500

H 7.034796 9.727320 7.588872

C 5.885381 7.963554 8.042465

H 5.204775 8.493943 8.704507

C 5.727879 6.576463 7.864296

C 3.800037 3.822580 9.633069

C 2.914627 5.054247 9.869879

C 3.119323 2.510806 9.462998

C 3.136778 1.545862 10.477887

H 3.666243 1.755985 11.409020

C 2.475865 0.325973 10.307754

H 2.496944 -0.419776 11.106596

C 1.784273 0.063305 9.122287

H 1.268240 -0.890658 8.987949

C 1.749863 1.028903 8.110121

H 1.207277 0.830540 7.182388

C 2.411449 2.244887 8.281067

H 2.397789 2.996173 7.487686

C 6.202623 3.347862 11.035300

H 5.918918 2.513063 11.682883

C 8.542626 6.916066 10.150003

H 9.466415 6.841652 9.577805

C 7.786280 5.758712 10.417703

C 8.241541 4.411165 9.991648

C 9.408617 4.237628 9.222746

H 9.959132 5.109542 8.870489

C 9.884089 2.973385 8.885765

H 10.787216 2.878760 8.278057

C 9.209953 1.832043 9.329434

H 9.579372 0.835468 9.076607

C 8.043027 1.979172 10.075089

H 7.484719 1.095922 10.396558

C 7.536005 3.247005 10.385353

C 2.456697 8.080044 10.435912

H 2.587925 7.499437 11.349866

C 1.855578 9.339641 10.485752

H 1.528841 9.745531 11.446623

No imaginary frequency.


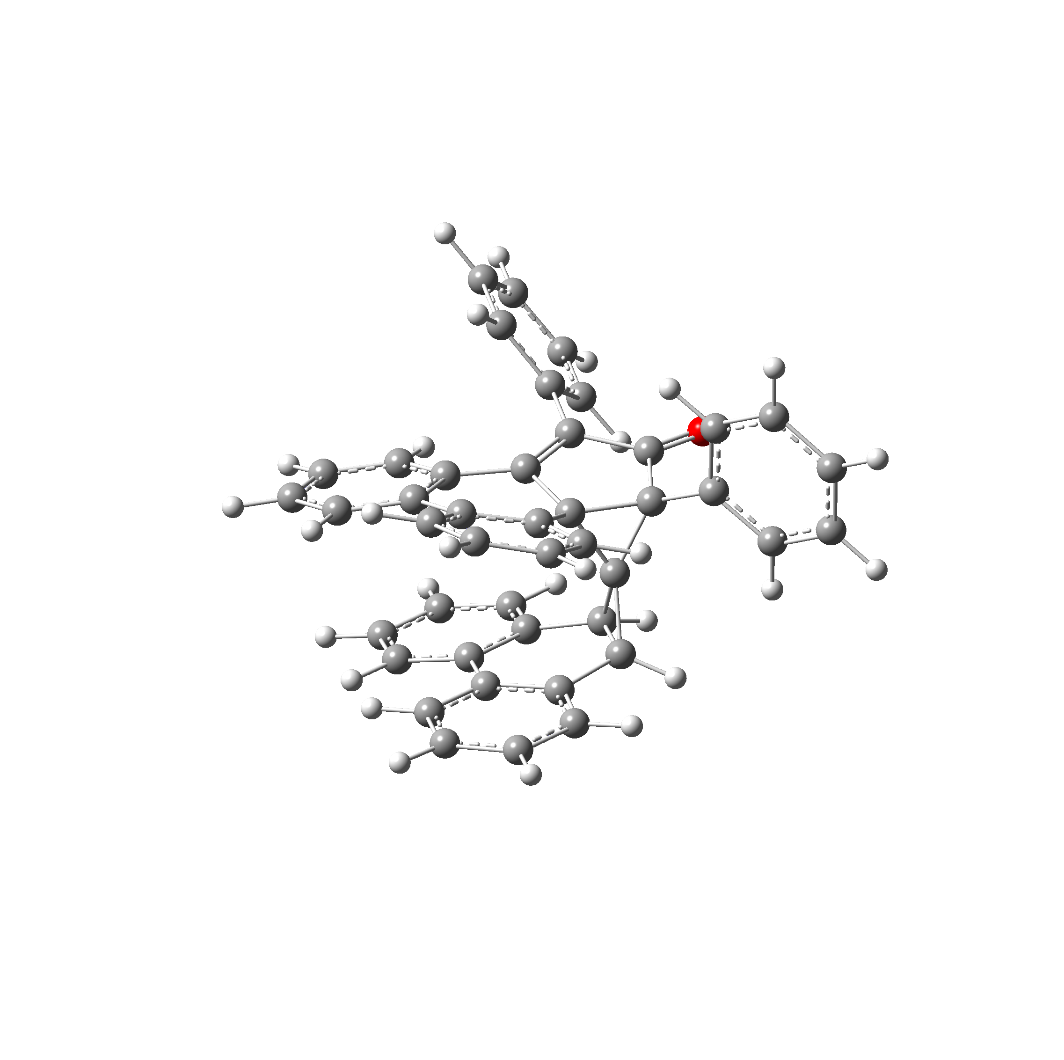


*Single point energies and T_1_ diagnostics for* ***9*** *and* ***8****; CCSD(T)/def2-TZVP//B2PLYP/def2-TZVP:*

**9**

------------------------- --------------------

FINAL SINGLE POINT ENERGY -1765.414311479760

------------------------- --------------------

T1 diagnostic ... 0.010634541

**8**

------------------------- --------------------

FINAL SINGLE POINT ENERGY -1765.424689847255

------------------------- --------------------

T1 diagnostic ... 0.010562023

*PES diagram for singlet* ***4*** *to allene* ***5***


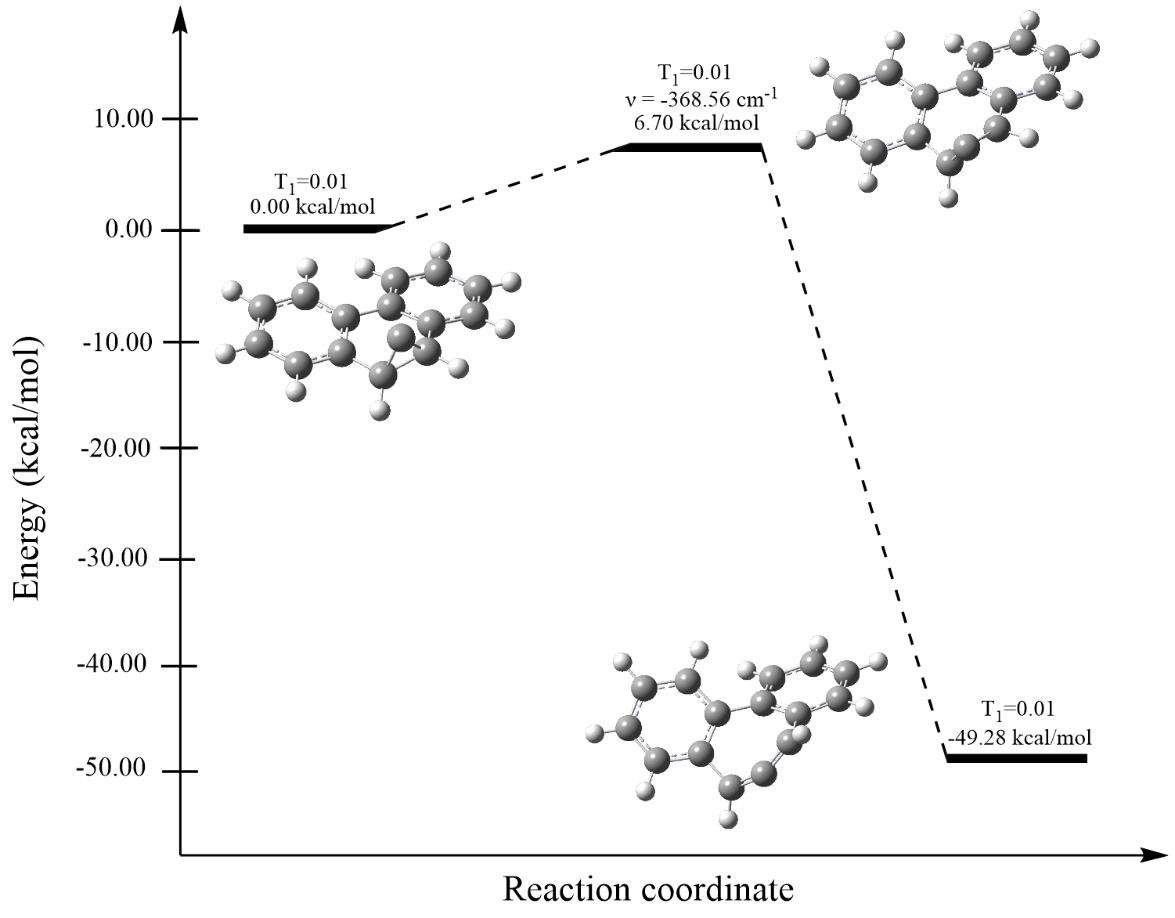


**4**

**TS**

**5**

**References**

1. Takeuchi, D.; Okada, T.; Kuwabara, J.; Osakada, K., Living Alternating Copolymerization of a Methylenecyclopropane Derivative with CO to Afford Polyketone with Dihydrophenanthrene-1,10-diyl Groups. *Macromolecular Chemistry and Physics* **2006,** *207*, 1546-1555.

2. Xu, L.; Bai, J.; Du, A.; Yang, Z.; Wu, B., 1,4-Diphenyltriphenylene grafted polysiloxane as a stationary phase for gas chromatography. *New Journal of Chemistry* **2020,** *44*, 695-703.

3. Menges, F. *Spectragryph - Optical Spectroscopy Software*, Version 1.2.15; 2020.

4. Bruker *Apex3*, Version 2019.11-0. ; Bruker AXS, Inc. Madison, Wisconsin, USA.2019.

5. Bruker *SAINT*, V. 8.37A. ; Bruker AXS, Inc. Madison, Wisconsin, USA.2015.

6. Krause, L.; Herbst-Irmer, R.; Sheldrick, G. M.; Stalke, D., Comparison of silver and molybdenum microfocus X-ray sources for single-crystal structure determination. *Journal of Applied Crystallography* **2015,** *48*, 3-10.

7. Dolomanov, O. V.; Bourhis, L. J.; Gildea, R. J.; Howard, J. A. K.; Puschmann, H., OLEX2: a complete structure solution, refinement and analysis program. *Journal of Applied Crystallography* **2009,** *42*, 339-341.

8. Sheldrick, G. M., Crystal structure refinement with SHELXL. *Acta Cryst. C* **2015,** *71*, 3-8.

9. Sheldrick, G., SHELXT - Integrated space-group and crystal-structure determination. *Acta Crystallographica Section A* **2015,** *71*, 3-8.

10. Neese, F.; Wennmohs, F.; Becker, U.; Riplinger, C., The ORCA quantum chemistry program package. *J. Chem. Phys.* **2020,** *152*, 224108.

11. Grimme, S., Semiempirical hybrid density functional with perturbative second-order correlation. *The Journal of Chemical Physics* **2006,** *124*, 034108.

12. Weigend, F.; Ahlrichs, R., Balanced basis sets of split valence, triple zeta valence and quadruple zeta valence quality for H to Rn: Design and assessment of accuracy. *Physical Chemistry Chemical Physics* **2005,** *7*, 3297-3305.

13. Weigend, F., Accurate Coulomb-fitting basis sets for H to Rn. *Physical Chemistry Chemical Physics* **2006,** *8*, 1057-65.

14. Hellweg, A.; Hättig, C.; Höfener, S.; Klopper, W., Optimized accurate auxiliary basis sets for RI-MP2 and RI-CC2 calculations for the atoms Rb to Rn. *Theoretical Chemistry Accounts* **2007,** *117*, 587-597.

15. Chmela, J.; Harding, M. E., Optimized auxiliary basis sets for density fitted post-Hartree–Fock calculations of lanthanide containing molecules. *Molecular Physics* **2018,** *116*, 1523-1538.

16. Becke, A. D., Density-functional exchange-energy approximation with correct asymptotic behavior. *Physical Review A* **1988,** *38*, 3098-3100.

17. Becke, A. D., Density‐functional thermochemistry. III. The role of exact exchange. *The Journal of Chemical Physics* **1993,** *98*, 5648-5652.

18. Riplinger, C.; Pinski, P.; Becker, U.; Valeev, E. F.; Neese, F., Sparse maps—A systematic infrastructure for reduced-scaling electronic structure methods. II. Linear scaling domain based pair natural orbital coupled cluster theory. *The Journal of Chemical Physics* **2016,** *144*, 024109.

19. Riplinger, C.; Sandhoefer, B.; Hansen, A.; Neese, F., Natural triple excitations in local coupled cluster calculations with pair natural orbitals. *The Journal of Chemical Physics* **2013,** *139*, 134101.

20. Riplinger, C.; Neese, F., An efficient and near linear scaling pair natural orbital based local coupled cluster method. *The Journal of Chemical Physics* **2013,** *138*, 034106.

21. Neese, F., An improvement of the resolution of the identity approximation for the formation of the Coulomb matrix. *Journal of Computational Chemistry* **2003,** *24*, 1740-1747.

22. Izsák, R.; Neese, F., An overlap fitted chain of spheres exchange method. *The Journal of Chemical Physics* **2011,** *135*, 144105.

23. Neese, F.; Wennmohs, F.; Hansen, A.; Becker, U., Efficient, approximate and parallel Hartree–Fock and hybrid DFT calculations. A ‘chain-of-spheres’ algorithm for the Hartree–Fock exchange. *Chemical Physics* **2009,** *356*, 98-109.

24. Perdew, J. P.; Burke, K.; Ernzerhof, M., Generalized Gradient Approximation Made Simple. *Physical Review Letters* **1996,** *77*, 3865-3868.

25. Grimme, S.; Ehrlich, S.; Goerigk, L., Effect of the damping function in dispersion corrected density functional theory. *Journal of Computational Chemistry* **2011,** *32*, 1456-1465.

26. Lee, T. J.; Taylor, P. R., A diagnostic for determining the quality of single‐reference electron correlation methods. *International Journal of Quantum Chemistry* **2009,** *36*, 199-207.

27. Zhurko, G. A.; Zhurko, D. A. *Chemcraft - graphical program for visualization of quantum chemistry computations.*: Ivanovo, Russia, 2005.
